# Enantioselective Hydrolysis of Amino Acid Esters Promoted by Bis(β-cyclodextrin) Copper Complexes

**DOI:** 10.1038/srep22080

**Published:** 2016-02-26

**Authors:** Shan-Shan Xue, Meng Zhao, Zhuo-Feng Ke, Bei-Chen Cheng, Hua Su, Qian Cao, Zhen-Kun Cao, Jun Wang, Liang-Nian Ji, Zong-Wan Mao

**Affiliations:** 1 MOE Key Laboratory of Bioinorganic and Synthetic Chemistry, School of Chemistry and Chemical Engineering, Sun Yat-sen University, Guangzhou 510275, P. R. China

## Abstract

It is challenging to create artificial catalysts that approach enzymes with regard to catalytic efficiency and selectivity. The enantioselective catalysis ranks the privileged characteristic of enzymatic transformations. Here, we report two pyridine-linked bis(β-cyclodextrin) (bisCD) copper(II) complexes that enantioselectively hydrolyse chiral esters. Hydrolytic kinetic resolution of three pairs of amino acid ester enantiomers (S_1_–S_3_) at neutral pH indicated that the “back-to-back” bisCD complex **CuL**^**1**^ favoured higher catalytic efficiency and more pronounced enantioselectivity than the “face-to-face” complex CuL^2^. The best enantioselectivity was observed for *N*-Boc-phenylalanine 4-nitrophenyl ester (S_2_) enantiomers promoted by **CuL**^**1**^, which exhibited an enantiomer selectivity of 15.7. We observed preferential hydrolysis of *L*-S_2_ by **CuL**^**1**^, even in racemic S_2_, through chiral high-performance liquid chromatography (HPLC). We demonstrated that the enantioselective hydrolysis was related to the cooperative roles of the intramolecular flanking chiral CD cavities with the coordinated copper ion, according to the results of electrospray ionization mass spectrometry (ESI-MS), inhibition experiments, rotating-frame nuclear Overhauser effect spectroscopy (ROESY), and theoretical calculations. Although the catalytic parameters lag behind the level of enzymatic transformation, this study confirms the cooperative effect of the first and second coordination spheres of artificial catalysts in enantioselectivity and provides hints that may guide future explorations of enzyme mimics.

Because of the cooperation of the catalytic centre and the second coordination sphere, a native metalloenzyme can achieve biological transformations with remarkable efficiency and enantioselectivity under mild conditions *in vivo*^1^,^2^. Over the years, significant efforts have been dedicated to the biomimetic study of metalloenzymes to understand their structures and functions and design analogues to mimic the native enzymes’ structures and functions^3^. In the past few decades, synthetic compounds with functions resembling those of, for example, hydrolases, oxidases, and reductases have been reported^4^,^5^,^6^,^7^,^8^. Remarkable progress has been made in mimicking the functions of the enzymes that mediate electron transfer with respective to the catalytic efficiency^9^,^10^. However, studies of hydrolase mimetics have lagged far behind, regarding to either catalytic efficiency or selectivity^5^,^7^,^8^,^11^. Specifically, in biomimetic chemistry, the rational design of enantioselective hydrolase mimics remains challenging.

Modified cyclodextrins (CDs) have been exploited to construct artificial enzymes since the 1970s, as introduced by Breslow^5^,^6^,^12^,^13^,^14^. Previous studies have indicated that CDs’ host-guest interactions could result in increased substrate binding affinity and cooperative binding between intramolecular CDs^15^,^16^,^17^. In fact, the chiral nature of the CD cavity, particularly the cooperative effects of CDs with coordinated metal ion, has been somewhat underestimated. Appropriately modified CDs can be used in enantioselective molecular binding studies^18^,^19^, and mediate chemical^20^,^21^,^22^ and photochemical^23^,^24^,^25^ enantio-differentiating reactions. CDs play an important role in preorganizing the guest molecules through the hydrophobic interactions. A number of studies have reported the use of unmodified or simply modified CD monomers without metal ions for the enantioselective deacylation of chiral esters^26^,^27^,^28^,^29^,^30^,^31^,^32^. However, the cooperative enantioselective catalytic effect arising from CDs’ intramolecular interactions with coordinated metal ions have rarely been reported, and the cooperative mechanism also remains unclear. We reported the construction of metallohydrolase mimics and superoxide dismutase mimics with CD domains^5^,^33^,^34^,^35^,^36^,^37^,^38^,^39^. The hydrolase activities were studied with carboxylic acid esters and phosphate esters as model, non-chiral substrates. In this study, chiral *t*-butyloxycarbonyl (**Boc**)-protected aromatic amino acid esters **S**_**1**_-**S**_**3**_ were chosen as substrates (Fig. 1). These substrates, which contain amino acid moieties, are biologically relevant than the previously used substrates and thus allowed us to evaluate the interactions of chiral CD cavities with naturally occurring substances. Two bisCD copper(II) complexes were utilized as enantioselective hydrolase mimics, denoted **CuL**^**1**^ (**L**^**1**^ = 2,6-bis(6-mono-amino-β-cyclodextrin-methyl)-pyridine, the “back-to-back” complex^39^) and **CuL**^2^ (**L**^**2**^ = 2,6-bis(3-mono-amino-β-cyclodextrin-methyl)-pyridine, the “face-to-face” complex^40^). The different CD orientations of the two mimics were studied in parallel to evaluate the effect of the chiral cavities on enantioselective catalysis. Electrospray ionization mass spectrometry (ESI-MS), an inhibition assay, rotating-frame nuclear Overhauser effect spectroscopy (ROESY), and theoretical calculations were performed to gain deeper insights into the underlying mechanism.

## Results

### Synthesis and characterization of **CuL** ^**1**^ and CuL^2^

The two copper(II) complexes, **CuL**^**1**^ and **CuL**^2^, were synthesized according to our previously reported procedures (see Supplementary Figs S1 and S2 for the ESI-MS results)^35^,^38^,^39^. Because of the relationship between coordination geometry and catalytic ability, we were particularly interested in probing the coordination geometries of the copper(II) centre by using electron paramagnetic resonance (EPR) spectroscopy, which were performed at 100 K in a frozen solution of water and dimethylsulfoxide (DMSO) (see Supplementary Fig. S3). As a result, *g*_//_ 2.25 and *A*_//_ 160 were obtained for **CuL**^**1**^, and *g*_//_ 2.26 and *A*_//_ 165 were obtained for **CuL**^2^, indicating a distorted square pyramidal coordination geometry at the copper(II) centre in both cases^41^,^42^. This proposed geometry was also supported by the UV-Vis spectra (Supplementary Fig. S4), which showed a broad absorption band at approximately 700 nm that was assigned to the *d*-*d* transitions^42^,^43^.

### Hydrolysis of Boc-protected amino acid esters

**Figure 2:**
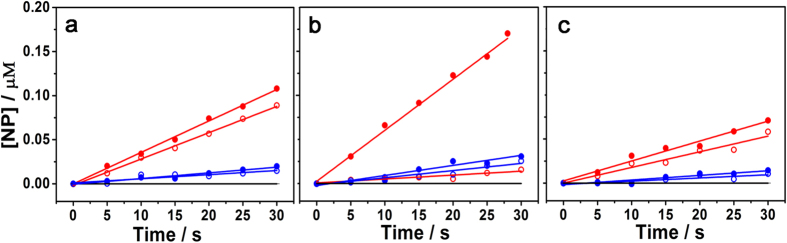
Initial-rate kinetics. Hydrolysis of single enantiomers of the substrates (2.5 μM) a) **S**_**1**_, b) **S**_**2**_, c) **S**_**3**_ by **CuL**^**1**^ (50 μM) (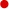 for the *L*-isomer, 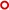 for the *D*-isomer) and **CuL**^2^ (50 μM) (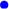 for the *L*-isomer, 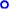 for the *D*-isomer) and spontaneous hydrolysis (the solid line in black). The reactions were performed in a 10% MeCN solution in HEPES buffer (pH 7.2, 50 mM) at 298 ± 0.1 K.

The hydrolysis of **S**_**1**_, **S**_**2**_ and **S**_**3**_ at neutral pH (50-mM 2-[4-(2-hydroxyethyl)piperazin-1-yl]ethanesulfonic acid (HEPES) buffer containing 10% CH_3_CN, pH 7.2) and 298 ± 0.1 K were monitored by UV-Vis spectroscopy, focusing on the absorption at 400 nm, which were proportional to the concentration of the hydrolytic product 4-nitrophenolate (**NP**). Figure 2 reveals that the concentration of **NP** (*ε*_obs_ = 10398 M^−1^ cm^−1^) varied as a function of time during the **CuL**^**1**^**-** and **CuL**^2^**-**promoted hydrolysis of **S**_**1**_, **S**_**2**_ and **S**_**3**_. The initial rate constants, *k*_in_ (s^−1^) of the substrate cleavage events are listed in Table 1. Accordingly, **CuL**^**1**^ exhibited a higher catalytic efficiency than **CuL**^2^. Moreover, the differences in the hydrolysis rates of the substrate enantiomers in the reactions with **CuL**^**1**^ were more pronounced than those in the reactions with **CuL**^2^. It should be noted that in the presence of **CuL**^**1**^ (50 μM), ***L*****-S**_**2**_ achieved the highest hydrolysis rate (*k*_in_^*L*^ = 5.4 × 10^−5^ s^−1^) among the tested substrates (Table 1), which was 168-fold higher than the spontaneous hydrolysis rate (*k*_uncat_ = 3.2 × 10^−7^ s^−1^) (2.5 μM) (Supplementary Fig. S12). In contrast, the **CuL**^**1**^**-**catalysed hydrolysis of ***D*****-S**_**2**_was 10-fold slower than that of ***L*****-S**_**2**_ under the same conditions (Fig. 2b). The enantioselectivity was reduced by changing either the substrate or the catalyst (Fig. 2a,c), while **S**_**2**_ remained the most favored substrate by both **CuL**^**1**^ and **CuL**^2^. Additionally, changing the catalyst reduced the enantioselectivity more than that induced by changing the substrate, suggesting that the CD orientation plays a very important role in the enantioselective catalysis. The results of these screening experiments indicated that the “back-to-back” bisCD complex **CuL**^**1**^ favoured the enantioselective hydrolysis of the amino acid-containing substrates, which was particularly evident for **S**_**2**_. The control experiments using the metal or ligands separately resulted in substantially decreased catalytic efficiency and enantioselectivity (Table 1 and Supplementary Figs S9–12).

**Figure 3:**
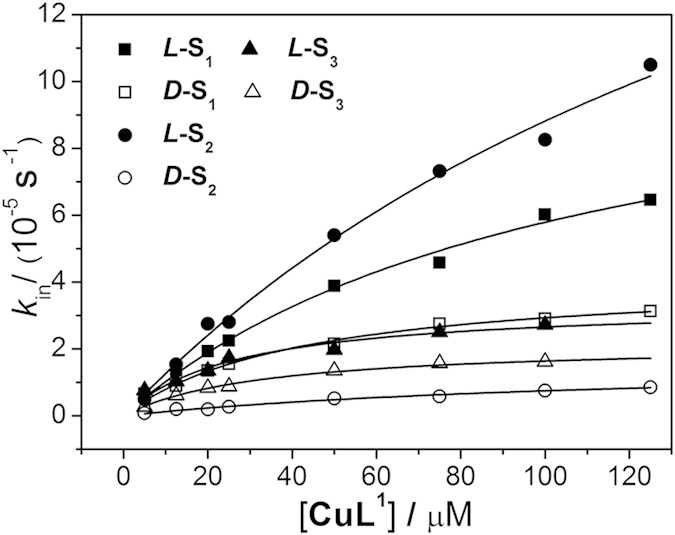
Saturation kinetics. Michaelis–Menten kinetics for the hydrolysis of the substrates (2.5 μM) in the presence of **CuL**^**1**^ (5.0–25 μM) in a 10% MeCN solution in HEPES buffer (pH 7.2, 50 mM) at 298 ± 0.1 K.

To fully assess the enantioselective hydrolysis of **S**_**1**_–**S**_**3**_ by **CuL**^**1**^, a revised Michaelis–Menten kinetic experiment was performed in the presence of excess catalyst^27^,^29^,^44^,^45^,^46^. By increasing the initial catalyst concentration from 5.0 to 125 μM, the hydrolysis rate increased, resulting in a levelled-off curve for each substrate (Fig. 3) and implying that the catalyst-substrate complex formed prior to the catalytic reaction. The kinetic parameters were deduced by fitting the data to the Michaelis–Menten equation (Table 2)^47^,^48^. The best catalytic ability (*k*_cat_/*k*_uncat_) was observed for ***L*****-S**_**2**_, which also showed the most pronounced enantioselectivity, with an enantiomer selectivity (*L*/*D*) of 15.7. Furthermore, the *k*_cat_ values for **S**_**1**_-**S**_**3**_ ranged from 3.3–6.2 × 10^−5^ s^−1^ for the *L*-isomers and 1.6–4.2 × 10^−5^ s^−1^ for the *D*-isomers. As a reference, the Michaelis–Menten kinetic experiment was also performed to investigate **S**_**2**_ catalysis by **CuL**^2^ (see Supplementary Fig. S14) and resulted in an enantiomer selectivity of 3.3, as shown in Supplementary Table S1.

To further confirm that the *L*-isomer was preferentially hydrolysed over the *D*-isomer, a chiral high-performance liquid chromatography (HPLC) analysis were performed with racemic **S**_**2**_. As shown in Supplementary Fig. S16, after different ratios of racemic **S**_**2**_ (10.0 μM) were consumed by **CuL**^**1**^ (100 μM) at 298 K, the reaction aliquots were subjected to ethyl acetate extraction to transfer the unreacted **S**_**2**_ into the organic phase^44^, followed by chiral HPLC analysis with CHIRALPAK^®^ IC. When 35%, 40%, and 50% of **S**_**2**_ was consumed, the remaining *L*:*D* ratios were 28:72, 23:77 and 20:80, respectively (Table 3). Based on these conversions and the remaining substrate enantiomeric ratios (e.r.), the consumption ratios of the ***L*****-S**_**2**_ and ***D*****-S**_**2**_ enantiomers were calculated to be 94:6, 92:8, and 82:18, thus indicating that the corresponding hydrolysed product (Boc-Phe-OH) were present at e.r. of 94:6, 92:8 and 82:18 (*L*/*D*) (Table 3). These results demonstrated that **CuL**^**1**^ possessed high enantioselectivity for the hydrolysis of racemic **S**_**2**_ and showed a preference for the *L*-isomers.

### Catalytic inhibition assay

**Figure 4:**
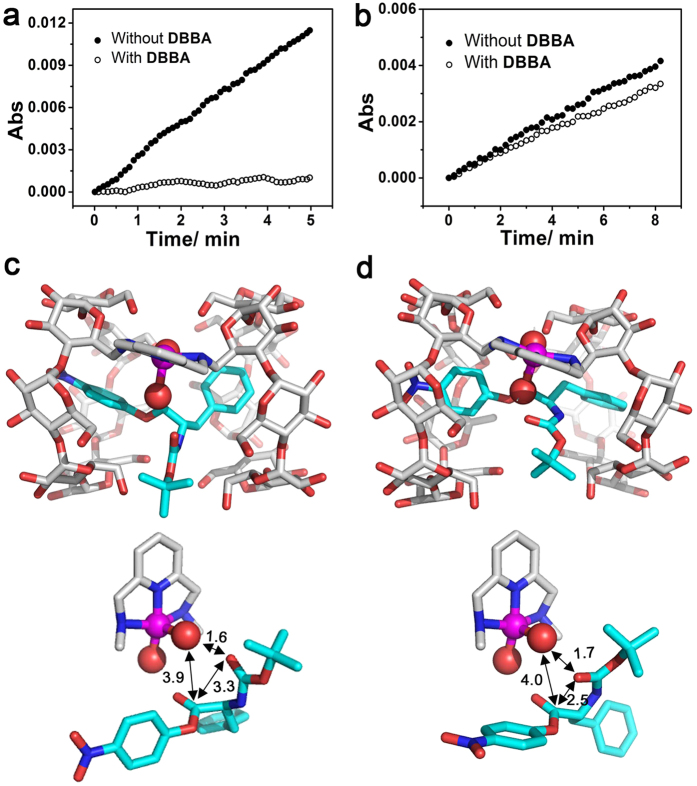
Inhibition reaction and theoretical calculation. Hydrolysis of (**a**) ***L*****-S**_**2**_, and (**b**) ***D*****-S**_**2**_ by **CuL**^**1**^ in the presence or absence of **DBBA** in HEPES buffer (pH 7.2, 50 mM) containing 10% MeCN at (298 ± 0.1) K, [**CuL**^**1**^] = [**S**_**2**_] = [**DBBA**] = 10.0 μM. Optimized structures of the (**c**) ***L*****-S**_**2**_-**CuL**^**1**^ and (**d**) ***D*****-S**_**2**_-**CuL**^**1**^ complexes at the ONIOM(B3LYP/lanl2dz:UFF)/IEFPCM level of theory. Color code: O, red; N, blue; Cu, magenta; C, light grey (catalysts) and cyan (substrates). Hydrogen was omitted. The intramolecular CDs were also omitted in the bottom images for clarity. The metal ions and coordinated water are shown as spheres. All labelled distances are shown in angstroms (Å).

To gain more insights into the reaction mechanism, di(*p*-*tert*-butylbenzyl) amine (**DBBA**) was applied as a CD inhibitor to investigate the role of the CD cavity^34^,^36^. In our previous study, we have excluded the possibility that **DBBA** exerts its inhibitory effect through coordination with the metal ions^36^. The strong binding between **DBBA** and the CD cavity was demonstrated by ROESY. The nuclear Overhauser effect (NOE) cross-peaks could be observed as the protons are closer than 0.4 nm in space. Therefore, the binding substituent group within the β-CD cavity could be estimated according to the relative intensity of the cross-peaks^49^. The interactions between the aryl protons of **DBBA** and the protons in the CD of **L**^**1**^ were observed (Supplementary Fig. S17). As expected, the initial rate of the **CuL**^**1**^-catalysed hydrolysis of ***L*****-S**_**2**_ was dramatically decreased by more than 13-fold in the presence of **DBBA**, suggesting that **CuL**^**1**^ was significantly inhibited (Fig. 4a,b). Although the hydrolysis rate for ***D*****-S**_**2**_ was low, a 1.3-fold decrease was still observed in the presence of **DBBA**. The kinetic parameters are displayed in Supplementary Table S2. Moreover, 2D ROESY nuclear magnetic resonance (NMR) experiments of **L**^**1**^ with *L*- or *D*- Boc-Phe-OH, instead of the **CuL**^**1**^ and **S**_**2**_ isomers, respectively, were performed (Supplementary Fig. S18). The ROESY spectrum of **L**^**1**^ with Boc-Phe-OH displayed NOE cross-peak signals between **H-3,5,6** of β-CD and the protons of the **Phenyl** and **Boc** groups of the Boc-Phe-OH enantiomers, indicating that the two groups could be self-included into the CD cavities of **L**^**1**^ from the primary side (Note: the binding constants (*K*_a_) by β-CD for benzene^50^ and *t*-BuOH^51^,^52^ are 120 M^−1^ and 48 M^−1^, respectively, at 298 K in H_2_O). These observations demonstrated that the hydrophobic CD cavities were indeed involved in the catalysis of both isomers.

ESI-MS analysis of the mixture of **CuL**^**1**^ with each **S**_**2**_ enantiomer were performed (see Supplementary Figs S19 and S20). The catalyst–substrate (1:1) complexes were detected by positive-ion ESI-MS, and no catalyst–product complexes were observed, thus indicating that the product binds the catalyst more weakly than the substrate. Considering the **NP** group could easily be bound in the β-CD cavity^38^,^53^, it leads to the proof that the substrate should be bound by the two cooperative hydrophobic cavities. Moreover, negative-ion ESI-MS confirmed that the hydrolysis products were *N*-Boc-phenylalanine (Boc-Phe-OH) and **NP**, and no Boc-deprotected product was observed. These observations suggested that there was enhanced affinity between the catalysts and substrates and that **NP** was cleaved from the substrates following the hydrolysis.

Based on the above results, we speculate that **NP** (*K*_a_ = 1503 M^−1^ (β-CD, 298 K, H_2_O))^54^ and the **Phenyl** groups of **S**_**2**_ were encapsulated in the cavities of **CuL**^**1**^ during the reaction. The enantioselectivity originated from the different geometries of the substrates that were regulated by the closely linked CD chiral cavities. The mechanism was further investigated through theoretical calculations. The structures of the ***L*****-S**_**2**_-**CuL**^**1**^ and ***D*****-S**_**2**_-**CuL**^**1**^ complexes were optimized at the ONIOM(B3LYP/lanl2dz:UFF)/IEFPCM level of theory (Fig. 4c,d). In both cases, the two aromatic side arms were encapsulated within the CD cavities, a result in good agreement with the results of the inhibition assay. The two isomers with different conformations were regulated by the CD chiral cavities. A major difference in the spatial distance between the ester carbonyl carbon and the flanking carbonyl oxygen was observed. In the case of ***L*****-S**_**2**_, the distance was 3.3 Å (Fig. 4c), and the ester carbonyl carbon was exposed to the metal-coordinated nucleophile. In the case of ***D*****-S**_**2**_, the flanking carbonyl oxygen was oriented toward the ester moiety, with a shorter distance of 2.5 Å (Fig. 4d), which probably hindered the nucleophilic attack from the metal-coordinated nucleophile because of possible negative charge repulsion. This difference should lead to the enantioselective hydrolysis of ***L*****-S**_**2**_ over ***D*****-S**_**2**_ by **CuL**^**1**^.

## Discussion

The initial-rate kinetic study showed that the “back-to-back” bisCD complex **CuL**^**1**^ exhibited higher catalytic efficiency and more pronounced enantioselectivity than the “face-to-face” analogue **CuL**^2^. These results indicated that the closely linked CD cavities on the primary face exhibit a much stronger cooperative effect with the coordinated copper ion in terms of differentiating the chiral substrates than those linked on the secondary face. Moreover, in most cases, the enantioselectivity was significantly decreased when only **L**^**1**^ or **L**^**2**^ was used without the metal. **S**_**3**_ enantiomers were an exception for **L**^**2**^, because containing the bulk R group, **S**_**3**_ would have an unsuitable location on **L**^**2**^ induced by the Cu^2+^ coordination with the bridge, as observed for the metal-inhibited enzymes^39^. Besides, in the presence of either **CuL**^**1**^ or **CuL**^2^, the initial hydrolysis rates of substrate *L*-isomers exceeded those of *D*-isomers in all cases, indicating that the chiral CD cavities in our models had an overall preference for the *L*-isomers of the amino acid esters. EtOH was tested as another cosolvent and exhibited an enantiomer selectivity similar to those of the reactions performed in MeCN. This finding indicated that a cosolvent effect of MeCN could be excluded (see Supplementary Fig. S13 and Table S4).

The *k*_cat_/*k*_uncat_ value of 811 for ***L*****-S**_**2**_ was in agreement with the largest values obtained for previously reported CD-based or other simple artificial mimics with similar substrates^26^,^27^,^28^,^55^,^56^, although some protein-based mimics produced higher values^44^,^57^,^58^,^59^. The most pronounced enantioselectivity was observed for the catalysis of **S**_**2**_ by **CuL**^**1**^, with an enantiomer selectivity of 15.7. This value is comparable to that of the reported natural protein-based model^44^. Although some reports have obtained better enantioselectivity with protein-modified catalysts, the complicated structure has made preparing the catalyst and performing mechanistic studies challenging^29^,^30^,^57^,^58^,^59^. The chiral HPLC analysis of **S**_**2**_ hydrolysis by **CuL**^**1**^ confirmed that the *L*-isomer was preferentially hydrolysed over ***D*****-S**_**2**_, with an e.r. of 94 :6 (*L*/*D*) when 35% of the racemic **S**_**2**_ was consumed. Finally, the **S**_**2**_ enantiomers were determined to be the optimal substrates for **CuL**^**1**^ in our study, and the removal of the methylene on the **Phenyl** group or replacement with an **Indolyl** group diminished the enantioselectivity. **CuL**^2^ also exhibited the highest enantioselectivity for **S**_**2**_compared with the other two pairs of enantiomers, although the cooperative effect was weaker than that of **CuL**^**1**^. Together, our results suggest that the substrate structure significantly affected the enantioselectivity, and **S**_**2**_ was identified as the best substrate among those tested. The findings indicated that the catalysts recognize specific substrates.

ESI-MS proved that the catalyst formed an intermediate complex with the substrates. The 2D ROESY NMR and inhibition assay demonstrated the vital role of CDs in the catalytic pathway. Finally, the optimized structures of the catalyst-substrate complexes provided initial evidence that the enantiomers formed different geometries according to the closely linked CD chiral cavities.

In conclusion, we presented a hydrolase mimetic study focusing on chiral substrates, in which two bisCD-based copper(II) complexes were developed for the hydrolysis of Boc-protected amino acid esters under physiological conditions (pH = 7.2). Accordingly, the adjacent chiral CD cavities had an overall preference for binding *L*-isomers. In addition, the “back-to-back” bisCD complex **CuL**^**1**^ exhibited much better catalytic efficiency and enantioselectivity towards the chiral amino acid esters than the “face-to-face” analogue **CuL**^2^. Mechanistic studies showed that the two closely linked chiral CD cavities played a vital role in mediating the enantioselective hydrolysis by regulating the different isomer geometries during the reaction, in which the hydrophobic groups of the substrate were embedded in the two intramolecular CD cavities. These findings indicate a cooperative effect of the first and second coordination spheres of artificial catalysts on the enantioselectivity and also provide hints that may guide future exploration of enzyme mimics.

## Methods

### Materials

Reagent-grade β-CD was recrystallized twice from H_2_O and dried in vacuo for 12 h at 373 K. All of the amino acid esters or precursor enantiomers, *N*-Boc-phenylalanine 4-nitrophenyl ester (**S**_**2**_), *N*-Boc-phenylglycine (Boc-Phg-OH), and *N*-Boc-Tryptophan (Boc-Trp-OH) were purchased from GL Biochem (Shanghai) Ltd. Dicyclohexylcarbodiimide (**DCC**) was purchased from Aladdin. Di(*p*-tert-butylbenzyl) amine (**DBBA**) was synthesized with previously reported methods^34^. DMF was superdry grade and stored over a molecular sieve. Common organic reagents were reagent grade and redistilled before use. Milli-Q water was used in all physical measurements. **CuL**^**1**^ and **CuL**^2^ were synthesized as described in previous studies^35^,^38^,^39^. All compounds were confirmed by elemental analyses, ESI-MS, and ^1^H-NMR spectra.

### General methods

The ^1^H NMR spectra were recorded on Mercury plus 300 spectrometers. The 2D NMR spectra were recorded on a Bruker AvanceIII 600 spectrometer. The elemental contents were analysed with a Perkin–Elmer 240 elemental analyser. The ESI-MS spectra were collected on a Thermo LCQ-DECA-XP spectrometer. The UV/Vis spectra were monitored with a Varian Cary 100 UV/Vis spectrophotometer equipped with a temperature controller (±0.1 K). The HPLC analyses were performed on an Agilent 1200 HPLC with CHIRALPAK^®^ IC 250 × 4.6 mm column. The EPR spectra were recorded on a Bruker A300-10-12 spectrometer. **Synthesis of the amino acid esters.** The amino acid esters were prepared according to a previously described method^60^, with some modifications. Boc-*L*-Phg-OH (3.5 g, 13.9 mmol) and 4-nitrophenol (1.9 g, 13.9 mmol) were dissolved in dry DMF (15 mL). Aliquots of the mixture were added to a stirred and cooled (−10 °C) suspension of **DCC** (2.9 g) in dry DMF (50 mL). The reaction mixture was magnetically stirred at −10 °C for approximately 2 h and then allowed to stand at room temperature for 5 h. The reaction mixture was filtered, and the filtrate was evaporated to dryness. The crystallization of the residue from 1:12 (*v*/*v*) ethyl acetate/petroleum ether produced pure Boc-*L*-Phg-ONp (***L*****-S**_**1**_) with a 78% yield. ^1^H-NMR (300 MHz, CDCl_3_): 8.22 (1H, d, *J* 9.2, NP-H), 7.42 (2H, dd, *J* 9.3, 3.6, Ph-H), 7.20 (1H, d, *J* 9.0, NP-H), 5.52 (1H, d, *J* 6.9, C-NH), 5.43 (1H, d, *J* 7.0, CH-N), 1.47 (4H, s, CH_3_); elemental analysis (calcd., observed for C_19_H_20_N_2_O_6_): C (61.28, 61.51), H (5.41, 5.44), N (7.52, 7.52).

***D*****-S**_**1**_ was prepared through the same procedure. ^1^H-NMR (300 MHz, CDCl_3_): 8.22 (1H, d, *J* 9.2, NP-H), 7.42 (2H, dd, *J* 9.3, 3.6, Ph-H), 7.20 (1H, d, *J* 9.0, NP-H), 5.52 (1H, d, *J* 6.9, C-NH), 5.43 (1H, d, *J* 7.0, CH-N), 1.47 (4H, s, CH_3_); elemental analysis (calcd., observed for C_19_H_20_N_2_O_6_): C (61.28, 60.94), H (5.41, 5.48), N (7.52, 7.52).

Boc-*L*-Trp-ONp (***L*****-S**_**3**_) and Boc-*D*-Trp-ONp (***D*****-S**_**3**_) were synthesized as described above.***L*****-S**_**3**_: ^1^H-NMR (300 MHz, CDCl_3_) 8.17 (3H, d, *J* 9.1, NP-H, Indole-H), 7.58 (1H, d, *J* 7.8, Indole-H), 7.40 (1H, d, *J* 8.1, Indole-H), 7.26–7.20 (3H, m, Indole-H, CHCl_3_), 7.17–7.08 (2H, m, NP-H), 6.98 (2H, d, *J* 9.1, Indole-H), 5.14 (1H, d, *J* 7.5, C-NH), 4.85 (1H, dd, *J* 13.5, 5.9, CH-N), 3.42 (2H, qd, *J* 14.3, 6.4, CH_2_), 1.45 (9H, s, CH_3_); elemental analysis (calcd., observed for C_22_H_23_N_3_O_6_): C (62.11, 62.34), H (5.45, 5.50), N (9.88, 9.77).

***D*****-S**_**3**_: ^1^H-NMR (300 MHz, CDCl_3_) 8.18 (3 H, d, *J* 9.0, NP-H, Indole-H), 7.60 (1 H, d, *J* 7.9, Indole-H), 7.40 (1 H, d, *J* 13.4, Indole-H), 7.29–7.21 (3 H, m, Indole-H, CHCl_3_), 7.13 (2 H, t, *J* 7.3, NP-H), 6.99 (2 H, d, *J* 9.0, Indole-H), 5.15 (1 H, d, *J* 7.7, C-NH), 4.86 (1 H, d, *J* 7.1, CH-N), 3.59–3.29 (2 H, m, CH_2_), 1.46 (9 H, s, CH_3_); elemental analysis (calcd., observed for C_22_H_23_N_3_O_6_): C (62.11, 62.11), H (5.45, 5.51), N (9.88, 9.81).

### Kinetics of amino acid ester hydrolysis

The hydrolysis rates of the amino acid esters in the presence of **CuL**^**1**^ and **CuL**^2^ were measured with an initial slope method by monitoring the increase in the 400-nm absorption of the released **NP**. At this wavelength, the absorbance of the ester substrate was negligible. The reaction solution was maintained at 298 ± 0.1 K. HEPES buffer (pH = 7.2, 50 mM) was used, and the ionic strength was adjusted to 0.10 M with NaClO_4_. The amino acid esters were prepared as solutions in CH_3_CN, and the buffer and catalyst solutions were freshly prepared in water.

In a typical experiment, a small amount of aqueous catalyst solution was spread evenly in a 600-μL cuvette containing 10% (*v*/*v*) CH_3_CN buffer solution, and the reactions were always initiated by injecting a small amount of substrate CH_3_CN solution, followed by complete mixing. All of the solutions were equilibrated to 298 ± 0.1 K. The initial first-order rate constants (*k*_in_ (s^−1^)) for the substrate cleavage were obtained directly from a plot of the **NP** concentration versus time by using the initial rate method. The errors in the *k*_in_ values were less than 5%. The *ε* value for **NP** at pH 7.2 was 10,398. The inhibition study was performed by following a similar procedure, except for the pre-equilibrium incubation after mixing **DBBA** with **CuL**^**1**^.

### Kinetic resolution of racemic S_2_

The hydrolysis of racemic **S**_**2**_ was performed using a method similar to that described in the previous section. Racemic **S**_**2**_ (10.0 μM) was mixed with **CuL**^**1**^ (100 μM) under the same conditions as those used in the kinetic studies. The reaction process was monitored with a UV/Vis spectrometer. Then, upon mixing of the reaction mixture with ethyl acetate, the products (Boc-Phe-OH and 4-nitrophenol) and remaining **S**_**2**_ were completely extracted into the organic phase^44^. The organic phase was evaporated to dryness under reduced pressure, and the resulting residue was dissolved in ethanol for HPLC analysis. The chiral HPLC analyses were performed at room temperature with a CHIRALPAK^®^ IC 250 × 4.6 mm column and monitored by a UV-Vis detector at 254 nm; the elution solvent was 40/30/30 H_2_O (0.1% formic acid)/CH_3_CN/EtOH, and the flow rate was 1.0 mL/min.

## Additional Information

**How to cite this article**: Xue, S.-S. *et al*. Enantioselective Hydrolysis of Amino Acid Esters Promoted by Bis(β-cyclodextrin) Copper Complexes. *Sci. Rep*. **6**, 22080; doi: 10.1038/srep22080 (2016).

## Supplementary Material

Supplementary Information

## Figures and Tables

**Figure 1:**
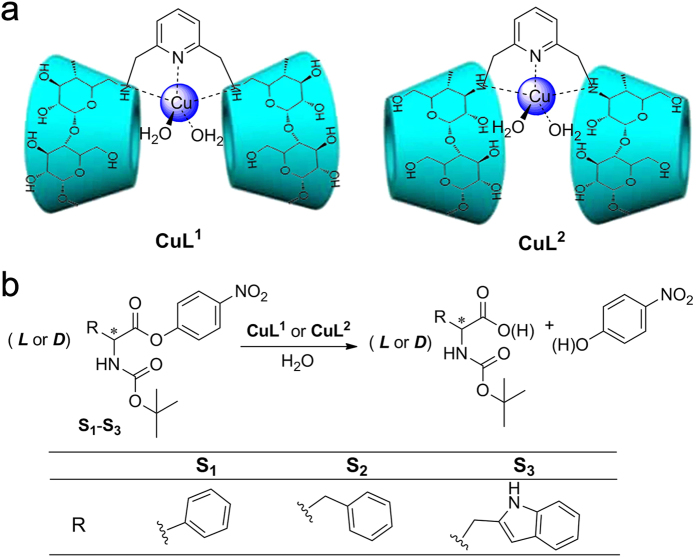
Schematic representations of (**a**) the structures of **CuL**^**1**^ and **CuL**^2^ and (**b**) the hydrolysis of **S**_**1**_, **S**_**2**_ and **S**_**3**_ catalysed by **CuL**^**1**^ and **CuL**^2^.

**Table 1:** The initial rate constants for S_1_-S_3_ (2.5 μM) promoted by different catalysts (50.0 μM) in a 10% MeCN solution in HEPES buffer (pH 7.2, 50 mM) at 298 ± 0.1 K.

Catalyst	*k*_in_ (10^−5^ s^−1^)					
	S_1_		S_2_		S_3_	
	*L*-	*D*-	*L*-	*D*-	*L*-	*D*-
**CuL**^**1**^	3.9	2.1	5.4	5.2 × 10^**−**1^	2.0	1.3
**CuL**^2^	9.5 × 10^**−**1^	7.7 × 10^**−**1^	1.6	7.2 × 10^**−**1^	3.4 × 10^**−**1^	1.9 × 10^**−**1^
**L**^**1**^	6.2 × 10^**−**1^	4.3 × 10^**−**1^	8.5 × 10^**−**1^	1.8 × 10^**−**1^	2.1 × 10^**−**1^	1.2 × 10^**−**1^
**L**^**2**^	4.4 × 10^**−**1^	3.1 × 10^**−**1^	4.8 × 10^**−**1^	1.2 × 10^**−**1^	4.3 × 10^**−**1^	1.7 × 10^**−**1^
**Cu**^**2+**^	1.2 × 10^**−**1^		6.7 × 10^**−**2^		6.1 × 10^**−**2^	
Buffer	5.0 × 10^**−**2^		3.2 × 10^**−**2^		2.8 × 10^**−**2^	

**Table 2:** Kinetic parameters for the hydrolysis of S_1_-S_3_ (2.5 μM) in the presence of CuL^1^ (5.0–125 μM) in a 10% MeCN solution in HEPES buffer (50 mM, pH = 7.2) at 298 ± 0.1 K.

		*k*_cat_ (10^−5^s^−1^)	*K*_m_(μM)	*k*_cat_/*K*_m_ (M^−1^ s^−1^)	*k*_uncat_ (10^−7^s^−1^)	*k*_cat_*/k*_uncat_	*k*_cat_^*L*^/*k*_cat_^*D*^
**S**_**1**_	***L-***	12.0 ± 1.0	107.0 ± 16.0	1.1	5.0	2.5 × 10^2^	2.9
	***D-***	4.2 ± 0.2	42.5 ± 4.3	1.0		8.4 × 10	
**S**_**2**_	***L-***	26.2 ± 4.2	197.0 ± 46.0	1.3	3.2	8.1 × 10^2^	15.7
	***D-***	1.7 ± 0.3	124.0 ± 32.0	0.1		5.2 × 10	
**S**_**3**_	***L-***	3.3 ± 0.3	28.5 ± 5.6	1.2	2.8	1.2 × 10^2^	1.5
	***D-***	2.2 ± 0.1	34.6 ± 3.4	0.6		7.9 × 10	

**Table 3:** Chiral HPLC analysis of the conversion of various amounts of racemic **S**.

Entry	Conversion (%)	Remaining e.r. of S_2_(*L*:*D*)^[a]^	e.r. of Boc-Phe-OH (*L*:*D*)^[b]^
1	35	28:72	94:6
2	40	23:77	92:8
3	50	20:80	82:18

[a] The enantiomeric ratio (e.r.) values of remaining substrates were determined by HPLC analysis with a chiral stationary phase. [b] e.r. values of products were calculated from the result of HPLC analysis.

## References

[b1] Hollmann F. & Otten L. G. Enantioselectivity of Enzymes in Wiley Encyclopedia of Chemical Biology (ed. Begley T.) (John Wiley & Sons, 2009).

[b2] Patel R. N. Biocatalysis: Synthesis of Key Intermediates for Development of Pharmaceuticals. ACS Catal. 1, 1056–1074 (2011).

[b3] Lu Y., Yeung N., Sieracki N. & Marshall N. M. Design of functional metalloproteins. Nature 460, 855–862 (2009). 19675646 10.1038/nature08304 PMC2770889

[b4] Dong Z., Luo Q. & Liu J. Artificial enzymes based on supramolecular scaffolds. Chem. Soc. Rev. 41, 7890–7908 (2012). 22972005 10.1039/c2cs35207a

[b5] Zhao M., Wang H.-B., Ji L.-N. & Mao Z.-W. Insights into metalloenzyme microenvironments: biomimetic metal complexes with a functional second coordination sphere. Chem. Soc. Rev. 42, 8360–8375 (2013). 23881282 10.1039/c3cs60162e

[b6] Rebilly J.-N., Colasson B., Bistri O., Over D. & Reinaud O. Biomimetic cavity-based metal complexes. Chem. Soc. Rev. 44, 467–489 (2015). 25319612 10.1039/c4cs00211c

[b7] Diez-Castellnou M., Mancin F. & Scrimin P. Efficient phosphodiester cleaving nanozymes resulting from multivalency and local medium polarity control. J. Am. Chem. Soc. 136, 1158–1161 (2014). 24405094 10.1021/ja411969e

[b8] Tirel E. Y., Bellamy Z., Adams H., Duarte F. & Williams N. H. Catalytic Zinc Complexes for Phosphate Diester Hydrolysis. Angew. Chem. Int. Ed. 53, 1–6 (2014). 10.1002/anie.201400335 PMC4140542 24919567

[b9] Helm M. L., Stewart M. P., Bullock R. M., DuBois M. R. & DuBois D. L. A synthetic nickel electrocatalyst with a turnover frequency above 100,000 s(-)(1) for H(2) production. Science 333, 863–866 (2011). 21836012 10.1126/science.1205864

[b10] Han Z., Qiu F., Eisenberg R., Holland P. L. & Krauss T. D. Robust photogeneration of H2 in water using semiconductor nanocrystals and a nickel catalyst. Science 338, 1321–1324 (2012). 23138979 10.1126/science.1227775

[b11] Mancin F., Scrimin P. & Tecilla P. Progress in artificial metallonucleases. Chem. Commun. 48, 5545–5559 (2012). 10.1039/c2cc30952a 22543403

[b12] Breslow R. & Overman L. E. An “Artificial Enzyme” Combining a Metal Catalytic Group and a Hydrophobic Binding Cavity. J. Am. Chem. Soc. 92, 1075–1077 (1970). 5451011 10.1021/ja00707a062

[b13] Li X. . Photocatalytic H2 production in aqueous solution with host-guest inclusions formed by insertion of an FeFe-hydrogenase mimic and an organic dye into cyclodextrins. Energ. Environ. Sci. 5, 8220–8224 (2012).

[b14] Watanabe K., Kitagishi H. & Kano K. Supramolecular iron porphyrin/cyclodextrin dimer complex that mimics the functions of hemoglobin and methemoglobin. Angew. Chem. Int. Ed. 52, 6894–6897 (2013). 10.1002/anie.201302470 23712820

[b15] Breslow R., Greenspoon N., Guo T. & Zarzycki R. Very Strong Binding of Appropriate Substrates by Cyclodextrin Dimers. J. Am. Chem. Soc. 111, 8297–8299 (1989).

[b16] Breslow R. & Zhang B. Very Fast Ester Hydrolysis by a Cyclodextrin Dimer with a Catalytic Linking Group. J. Am. Chem. Soc. 114, 5882–5883 (1992).

[b17] Liu Y. & Chen Y. Cooperative Binding and Multiple Recognition by Bridged Bis(*β*-cyclodextrin)s with Functional Linkers. Acc. Chem. Res. 39, 681–691 (2006). 17042468 10.1021/ar0502275

[b18] Wang H. . Diastereomeric Molecular Recognition and Binding Behavior of Bile Acids by L/D-Tryptophan-Modified *β*-Cyclodextrins. J. Org. Chem. 70, 8703–8711 (2005). 16238298 10.1021/jo051073+

[b19] Liu Y. . Molecular Recognition Study on a Supramolecular System. 10. Inclusion Complexation of Modified β-Cyclodextrins with Amino Acids: Enhanced Enantioselectivity for L/D-Leucine. J. Org. Chem. 62, 1826–1830 (1997).

[b20] Kanagaraj K., Suresh P. & Pitchumani K. Per-6-amino-β-cyclodextrin as a Reusable Promoter and Chiral Host for Enantioselective Henry Reaction. Org. Lett. 12, 4070–4073 (2010). 20731369 10.1021/ol101658n

[b21] Hu S. . Asymmetric Supramolecular Primary Amine Catalysis in Aqueous Buffer: Connections of Selective Recognition and Asymmetric Catalysis. J. Am. Chem. Soc. 132, 7216–7228 (2010). 20433173 10.1021/ja102819g

[b22] Schlatter A., Kundu M. K. & Woggon W.-D. Enantioselective reduction of aromatic and aliphatic ketones catalyzed by ruthenium complexes attached to beta-cyclodextrin. Angew. Chem. Int. Ed. 43, 6731–6734 (2004). 10.1002/anie.200460102 15593139

[b23] Lu R. . Enantiodifferentiating Photoisomerization of Cyclooctene Included and Sensitized by Aroyl-β-cyclodextrins: A Critical Enantioselectivity Control by Substituents. J. Org. Chem. 73, 7695–7701 (2008). 18759483 10.1021/jo801439n

[b24] Yao J. . Ammonia-Driven Chirality Inversion and Enhancement in Enantiodifferentiating Photocyclodimerization of 2-Anthracenecarboxylate Mediated by Diguanidino-gamma-cyclodextrin. J. Am. Chem. Soc. 136, 6916–6919 (2014). 24800988 10.1021/ja5032908

[b25] Nakamura A. & Inoue Y. Electrostatic Manipulation of Enantiodifferentiating Photocyclodimerization of 2-Anthracenecarboxylate within *γ*-Cyclodextrin Cavity through Chemical Modification. Inverted Product Distribution and Enhanced Enantioselectivity. J. Am. Chem. Soc. 127, 5338–5339 (2005). 15826169 10.1021/ja050704e

[b26] Hamasaki K. & Ueno A. Significant Enantioselectivity in Alanine Ester Hydrolysis Catalyzed by Imidazole Attached *β*-cyclodextrin. Chem. Lett. 859–860 (1995).

[b27] Goto K. . Cyclodextrin-Mediated Deacylation of Amino Acid Esters with Marked Stereoselectivity. Chem. Pharm. Bull. 50, 1283–1285 (2002). 12237555 10.1248/cpb.50.1283

[b28] Tsutsumi H., Hamasaki K., Mihara H. & Ueno A. Rate enhancement and enantioselectivity in ester hydrolysis catalysed by cyclodextrin–peptide hybrids. J. Chem. Soc., Perkin Trans. 2, 1813–1818 (2000).

[b29] Ueoka R. . Cyclodextrin-Mediated Deacylation of Peptide Esters with Marked Stereoselectivity. J. Am. Chem. Soc. 114, 8339–8340 (1992). 10.1248/cpb.50.1283 12237555

[b30] Breslow R., Trainor G. & Ueno A. Optimization of Metallocene Substrates for *β*-Cyclodextrin Reactions. J. Am. Chem. Soc 105, 2139–2144 (1983).

[b31] Easton C. J. & Lincoln S. F. Chiral Discrimination by Modified Cyclodextrins. Chem. Soc. Rev. 25, 163–170 (1996).

[b32] Beyrich T., Jira T. & Beyer C. Enantioselective Influence of Cyclodextrins on Cleavage of Chiralic Esters. Chirality 7, 560–564 (1995).

[b33] Fu H., Zhou Y.-H., Ji L.-N. & Mao Z.-W. Complexation, Structure, and Superoxide Dismutase Activity of the Imidazolate-Bridged Dinuclear Copper Moiety with *β*-Cyclodextrin and Its Guanidinium-Containing Derivative. J. Am. Chem. Soc. 128, 4924–4925 (2006). 16608305 10.1021/ja057717c

[b34] Zhou Y.-H., Zhao M., Mao Z.-W. & Ji L.-N. Ester hydrolysis by a cyclodextrin dimer catalyst with a metallophenanthroline linking group. Chem.-Eur. J. 14, 7193–7201 (2008). 18601233 10.1002/chem.200800237

[b35] Tang S.-P. . Ester hydrolysis by a cyclodextrin dimer catalyst with a tridentate N,N′,N″-zinc linking group. Chem. Asian. J. 4, 1354–1360 (2009). 19579255 10.1002/asia.200900108

[b36] Zhao M. . Effect of hydrophobic interaction cooperating with double Lewis acid activation in a zinc(II) phosphodiesterase mimic. Chem. Commun. 46, 6497–6499 (2010). 10.1039/c0cc00838a 20697618

[b37] Zhao M. . Unexpected phosphodiesterase activity at low pH of a dinuclear copper-beta-cyclodextrin complex. Chem. Commun. 47, 7344–7346 (2011). 10.1039/c1cc12466h 21643561

[b38] Tang S.-P. . Ester catalytic hydrolysis by a tridentate N,N′,N″-copper bridged cyclodextrin dimer. Inorg. Chem. Commun. 14, 184–188 (2011).

[b39] Hu P., Liu G.-F., Ji L.-N. & Mao Z.-W. Efficient promotion of phosphate diester cleavage by a face-to-face cyclodextrin dimer without metal. Chem. Commun. 48, 5515–5517 (2012). 10.1039/c2cc31490h 22538257

[b40] Chiu S.-H., Myles D. C., Garrell R. L. & Stoddart J. F. Novel Ether-Linked Secondary Face-to-Face 2-2′ and 3-3′ *β*-Cyclodextrin Dimers. J. Org. Chem. 65, 2792–2796 (2000). 10808457 10.1021/jo9910381

[b41] Hegg E. L. . Structure-Reactivity Studies in Copper(II)-Catalyzed Phosphodiester Hydrolysis. Inorg. Chem. 38, 2961–2968 (1999). 11671046 10.1021/ic981087g

[b42] Sengupta S. & Mondal R. Elusive nanoscale metal-organic-particle-supported metallogel formation using a nonconventional chelating pyridine-pyrazole-based bis-amide ligand. Chem.-Eur. J. 19, 5537–5541 (2013). 23508746 10.1002/chem.201204242

[b43] Castillo I. . Structural, spectroscopic, and electrochemical properties of tri- and tetradentate N3 and N3S copper complexes with mixed benzimidazole/thioether donors. Dalton. Trans. 41, 9394–9404 (2012). 22735464 10.1039/c2dt30756a

[b44] Tomisaka K., Ishida Y., Konishi K. & Aida T. Enantioselective hydrolysis of amino acid esters by apomyoglobin: perfect kinetic resolution of a phenylalanine derivative. Chem. Commun., 133–134 (2001).

[b45] Boseggia E. . Toward Efficient Zn(II)-Based Artificial Nucleases. J. Am. Chem. Soc. 126, 4543–4549 (2004). 15070372 10.1021/ja039465q

[b46] Mohamed M. F. & Brown R. S. Cleavage of an RNA model catalyzed by dinuclear Zn(II) complexes containing rate-accelerating pendants. Comparison of the catalytic benefits of H-bonding and hydrophobic substituents. J. Org. Chem. 75, 8471–8477 (2010). 21087029 10.1021/jo1017316

[b47] Zulkefeli M. . Selective hydrolysis of phosphate monoester by a supramolecular phosphatase formed by the self-assembly of a bis(Zn(2 + )-cyclen) complex, cyanuric acid, and copper in an aqueous solution (cyclen = 1,4,7,10-tetraazacyclododecane). Inorg. Chem. 50, 10113–10123 (2011). 21936489 10.1021/ic201072q

[b48] Arora H., Barman S. K., Lloret F. & Mukherjee R. Isostructural dinuclear phenoxo-/acetato-bridged manganese(II), cobalt(II), and zinc(II) complexes with labile sites: kinetics of transesterification of 2-hydroxypropyl-p-nitrophenylphosphate. Inorg. Chem. 51, 5539–5553 (2012). 22536852 10.1021/ic201971t

[b49] Liu Y., Shi J. & Guo D.-S. Novel Permethylated *β*-Cyclodextrin Derivatives Appended with Chromophores as Efficient Fluorescent Sensors for the Molecular Recognition of Bile Salts. J. Org. Chem. 72, 8227–8234 (2007). 17914840 10.1021/jo071131m

[b50] Sanemasa I. & Akamine Y. Association of benzene and alkylbenzenes with cyclodextrins in aqueous medium. Bull. Chem. Soc. Jpn. 60, 2059–2060 (1987).

[b51] Matsui Y. & Mochida K. Binding forces contributing to the association of cyclodextrin to alcohol in an aqueous solution. Bull. Chem. Soc. Jpn 52, 2808–2814 (1979).

[b52] Nakamura T., Takashima Y., Hashidzume A., Yamaguchi H. & Harada A. A metal-ion-responsive adhesive material via switching of molecular recognition properties. Nat. Commun. 5, 1–9 (2014). 10.1038/ncomms5622 PMC4143919 25099995

[b53] Ortega-Caballero F., Rousseau C., Christensen B., Petersen T. E. & Bols M. Remarkable Supramolecular Catalysis of Glycoside Hydrolysis by a Cyclodextrin Cyanohydrin. J. Am. Chem. Soc. 127, 3238–3239 (2005). 15755115 10.1021/ja042678a

[b54] Penn S. G. . Capillary Electrophoresis as a Method for Determining Binding Constants: Application to the Binding of Cyclodextrins and Nitrophenolates. J. Phys. Chem. 99, 3875–3880 (1995).

[b55] You J.-S. . Hydrolytic metalloenzyme models. J. Mol. Catal. A: Chem. 202, 17–22 (2003).

[b56] Scrimin P., Tecilla P. & Tonellato U. Chiral Lipophilic Ligands. 1. Enantioselective Cleavage of α-Amino Acid Esters in Metallomicellar Aggregates. J. Org. Chem. 59, 4194–4201 (1994).

[b57] Tanaka F., Kinoshita K., Tanimura R. & Fujii I. Relaxing Substrate Specificity in Antibody-Catalyzed Reactions: Enantioselective Hydrolysis of *N*-Cbz-Amino Acid Esters. J. Am. Chem. Soc. 118, 2332–2339 (1996).

[b58] Yano Y. . Fairly Marked Enantioselectivity for the Hydrolysis of Amino Acid Esters by Chemically Modified Enzymes. J. Org. Chem. 68, 1314–1318 (2003). 12585870 10.1021/jo0265075

[b59] Engström K., Nyhlén J., Sandström A. G. & Bäckvall J.-E. Directed Evolution of an Enantioselective Lipase with Broad Substrate Scope for Hydrolysis *α*-Substituted Esters. J. Am. Chem. Soc. 132, 7038–7042 (2010). 20450151 10.1021/ja100593j

[b60] D’Alagni M., Bemporad P. & Garofolo A. Sequence peptide polymers: Part 1. Poly(leucyl-leucyl-aspartic acid-*β*-benzyl ester)−synthesis and some conformational aspects in solutions. Polymer 13, 419–422 (1972).

