# Supplementary material for: Enantioselective Hydrolysis of Amino Acid Esters Promoted by Bis(β-cyclodextrin) Copper Complexes
Source: Sci Rep. 2016 Feb 26;6:22080. doi: 10.1038/srep22080 (PMC4768151; doi:10.1038/srep22080)
Supplement: Supplementary Information [file srep22080-s1.doc]

**Supplementary Information**

**Enantioselective Hydrolysis of Amino Acid Esters Promoted by Bis(β-cyclodextrin) Copper Complexes**

Shan-Shan Xue, Meng Zhao, Zhuo-Feng Ke, Bei-Chen Cheng, Hua Su, Qian Cao, Zhen-Kun Cao, Jun Wang*, Liang-Nian Ji and Zong-Wan Mao*

MOE Key Laboratory of Bioinorganic and Synthetic Chemistry

School of Chemistry and Chemical Engineering

Sun Yat-sen University

Guangzhou 510275 (P. R. China)

E-mail: wangjun23@mail.sysu.edu.cn; cesmzw@mail.sysu.edu.cn.

**Table of Content**

Figure S1. ESI-MS of **CuL^1^**. **3**

Figure S2. ESI-MS of **CuL^2^**. **3**

Figure S3. EPR spectra of **CuL^1^** and **CuL^2^**. **4**

Figure S4. UV/Vis spectra of Cu(ClO_4_)_2_, **L^1^**, **L^2^** and **CuL^1^**, **CuL^2^**.  **4**

Figure S5-8. ^1^H NMR spectrum of **S_1_** and **S_3_** enantiomers. **5**

Figure S9-11. Initial hydrolysis for **S_1_-S_3_** promoted by different catalysts. **7**

Figure S12. Values of *k*_in_/*k*_uncat_ for **S_1_**-**S_3_** promoted by different catalysts. **8**

Figure S13. Hydrolysis of **S_2_** catalyzed by **CuL^1^** with EtOH as the cosolvent. **9**

Figure S14. Michaelis–Menten kinetics for **S_2_** promoted by **CuL^2^**. **10**

Figure S15. HPLC results of ***L*-S_2_** and ***D*-S_2_**.  **10**

Figure S16. Chiral HPLC analyses for **S_2_** enantiomers in different conversions. **11**

Figure S17. ROESY spectrum of **L^1^** with **DBBA**.  **12**

Figure S18. ROESY spectrum of **L^1^** with Boc-Phe-OH.  **13**

Figure S19. ESI-MS spectra of mixed solution of **CuL^1^** and ***L*-S_2_**.  **14**

Figure S20. ESI-MS spectra of mixed solution of **CuL^1^** and ***D*-S_2_**.  **15**

Table S1. Kinetic parameters for **S_2_** catalyzed by **CuL^2^**. **16**

Table S2. Kinetic parameters of **CuL^1^** to **S_2_** enantiomers with or without **DBBA**. **16**

Table S3. Initial rate constants for **S_2_** promoted by different complexes.  **16**

Table S4. Initial rate constants for **S_2_** promoted by **CuL^1^** with different cosolvent.  **16**


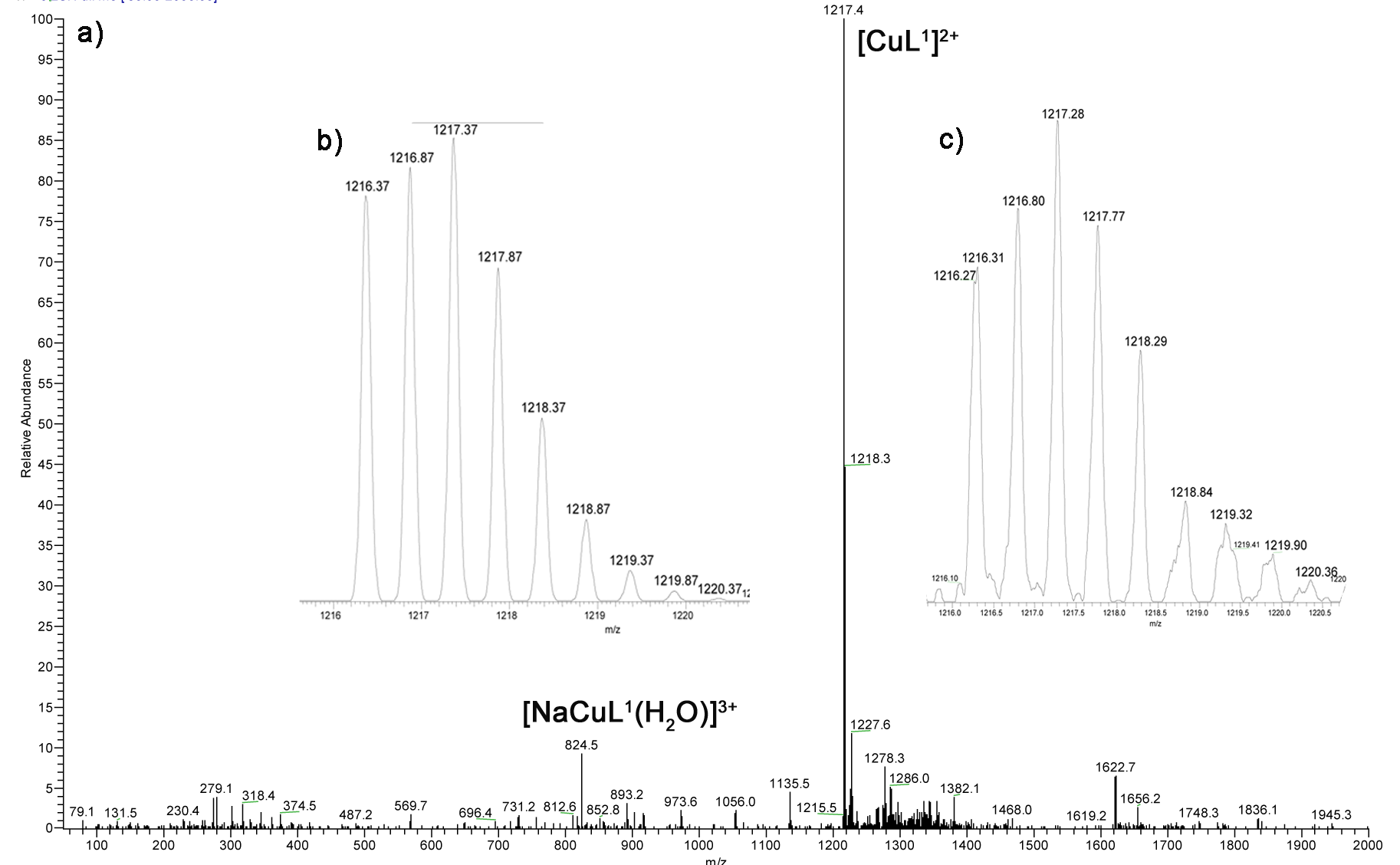


**Figure S1**. ESI-MS of **CuL^1^**: a) Full range spectra; b) Bivalence ion isotopes spectra of computer simulation using formula C_91_H_147_N_3_O_68_Cu, which is equivalent with [**CuL^1^**]^2+^; c) Bivalence ion isotopes spectra detected.


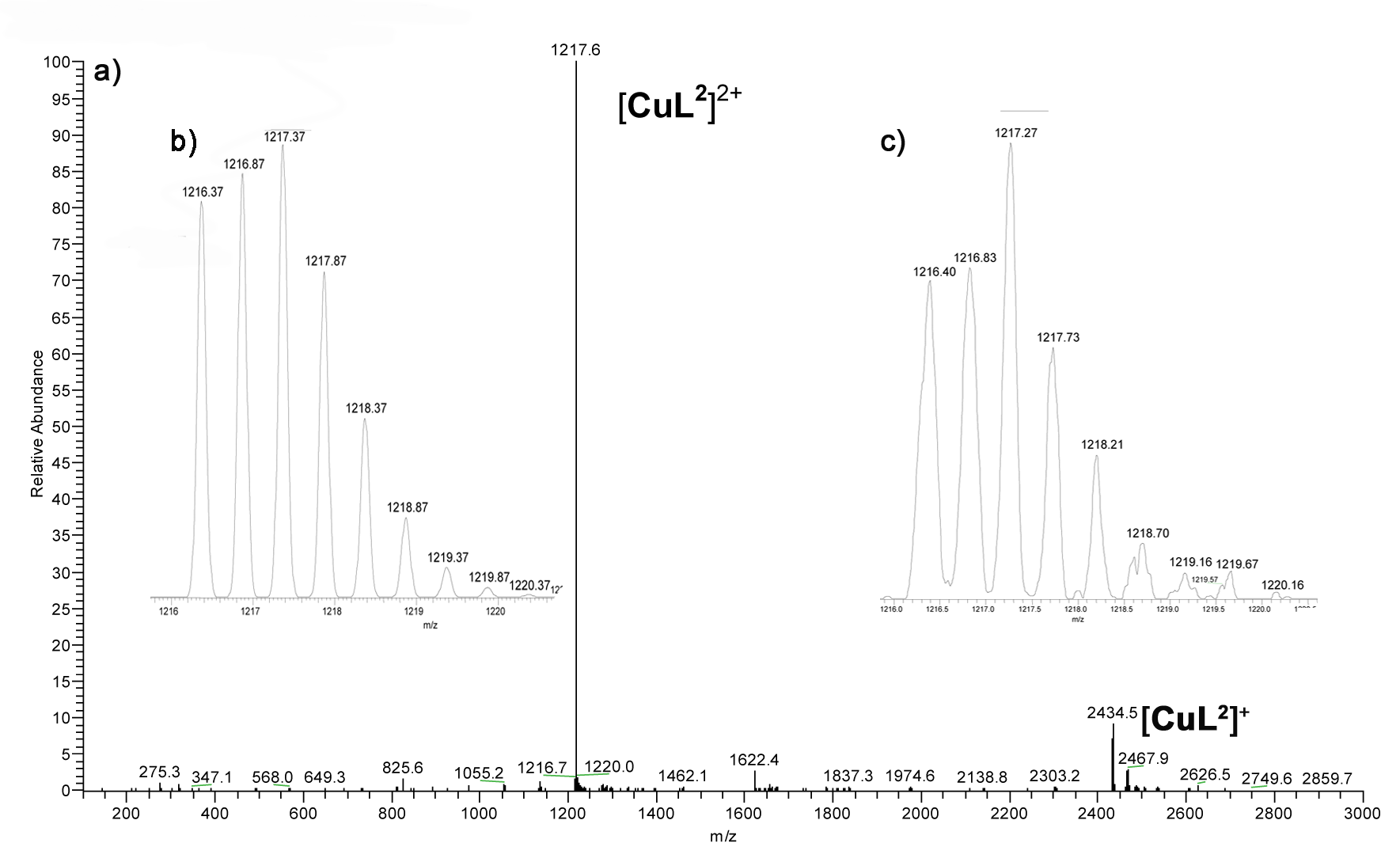


**Figure S2**. ESI-MS of **CuL^2^**: a) Full range spectra; b) Bivalence ion isotopes spectra of computer simulation using formula C_91_H_147_N_3_O_68_Cu, which is equivalent with [**CuL^2^**]^2+^; c) Bivalence ion isotopes spectra detected.


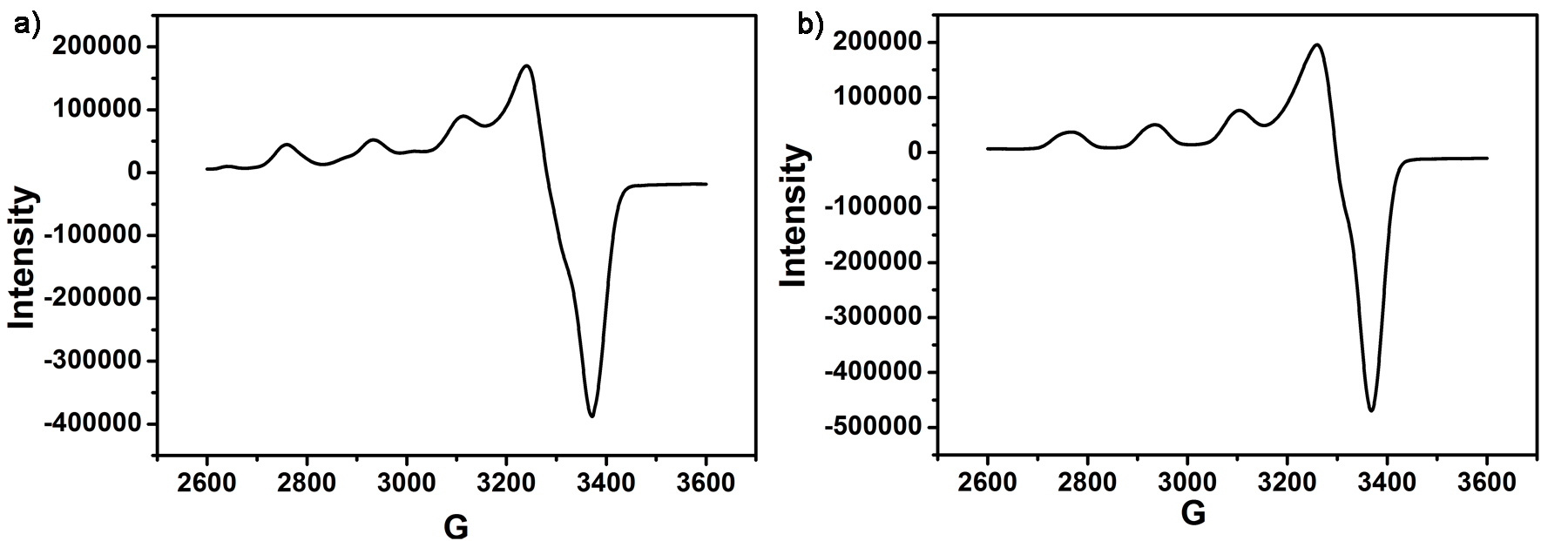


**Figure S3**. The frozen solution EPR spectra of a) **CuL^1^**, b) **CuL^2^**, performed in H_2_O/DMSO mixture solution at 100 K. Resulted parameters: *g*_∥_ = 2.25, *g*_⊥_ = 2.04, *A*// = 160 for **CuL^1^**, and *g*_∥_ = 2.26, *g*_⊥_ = 2.04, *A*// = 165 for **CuL^2^**.


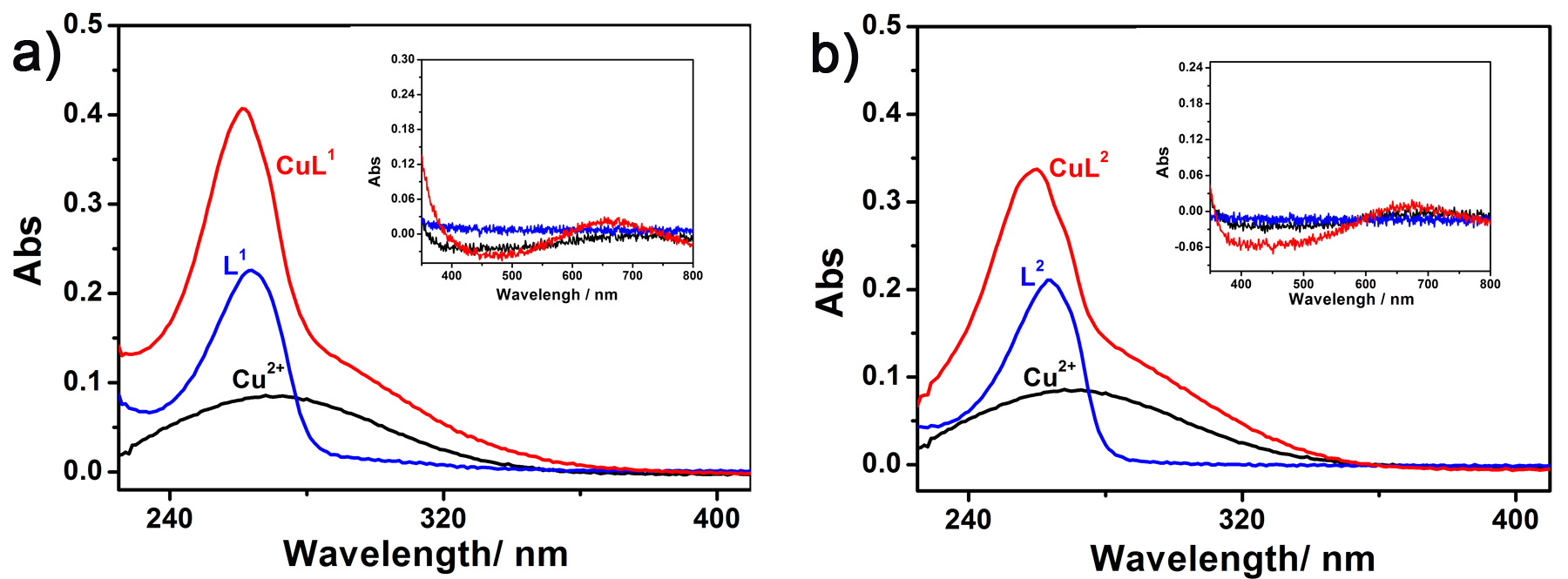


**Figure S4**. UV/Vis spectra of a) Cu(ClO_4_)_2_ (black), **L^1^** (blue), and **CuL^1^** (red) and b) Cu(ClO_4_)_2_ (black), **L^2^** (blue), and **CuL^2^** (red) in HEPES buffer (50 mM, pH 7.2) at (298 ± 0.1) K, with the concentration 0.04 mM.


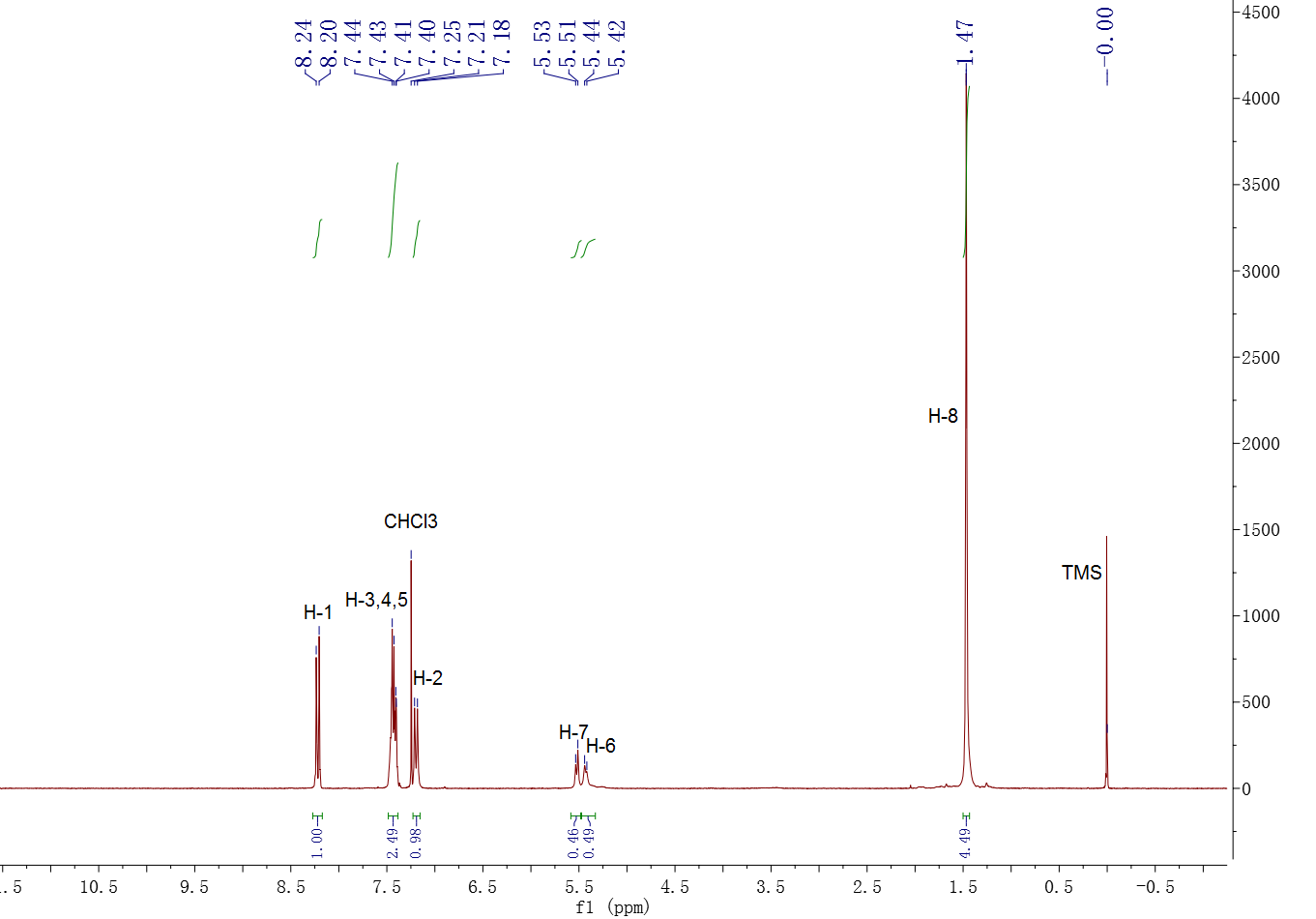


**Figure S5**. ^1^H NMR spectrum of ***L*-S_1_** in CDCl_3_.


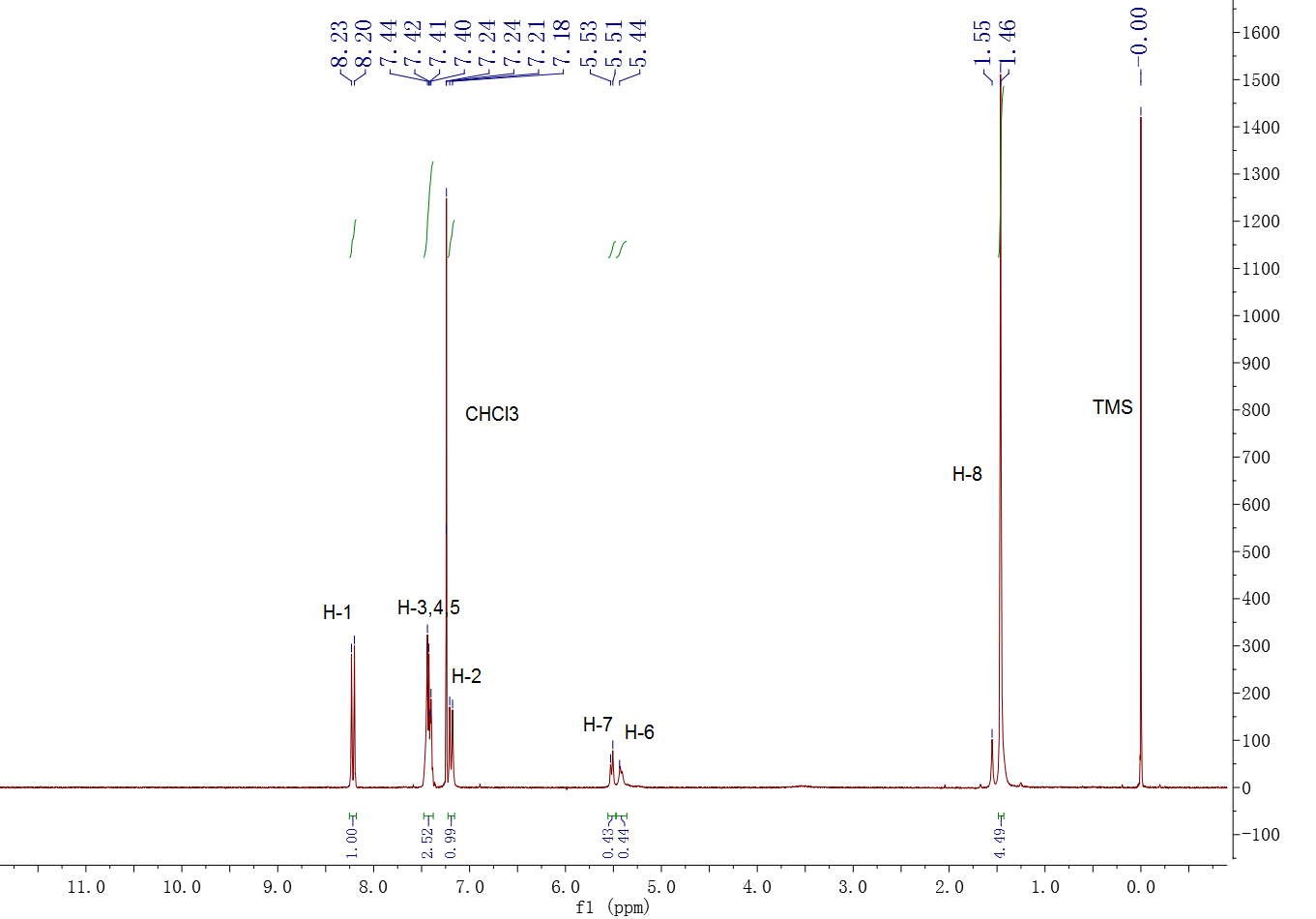


**Figure S6**. ^1^H NMR spectrum of ***D*-S_1_** in CDCl_3_.


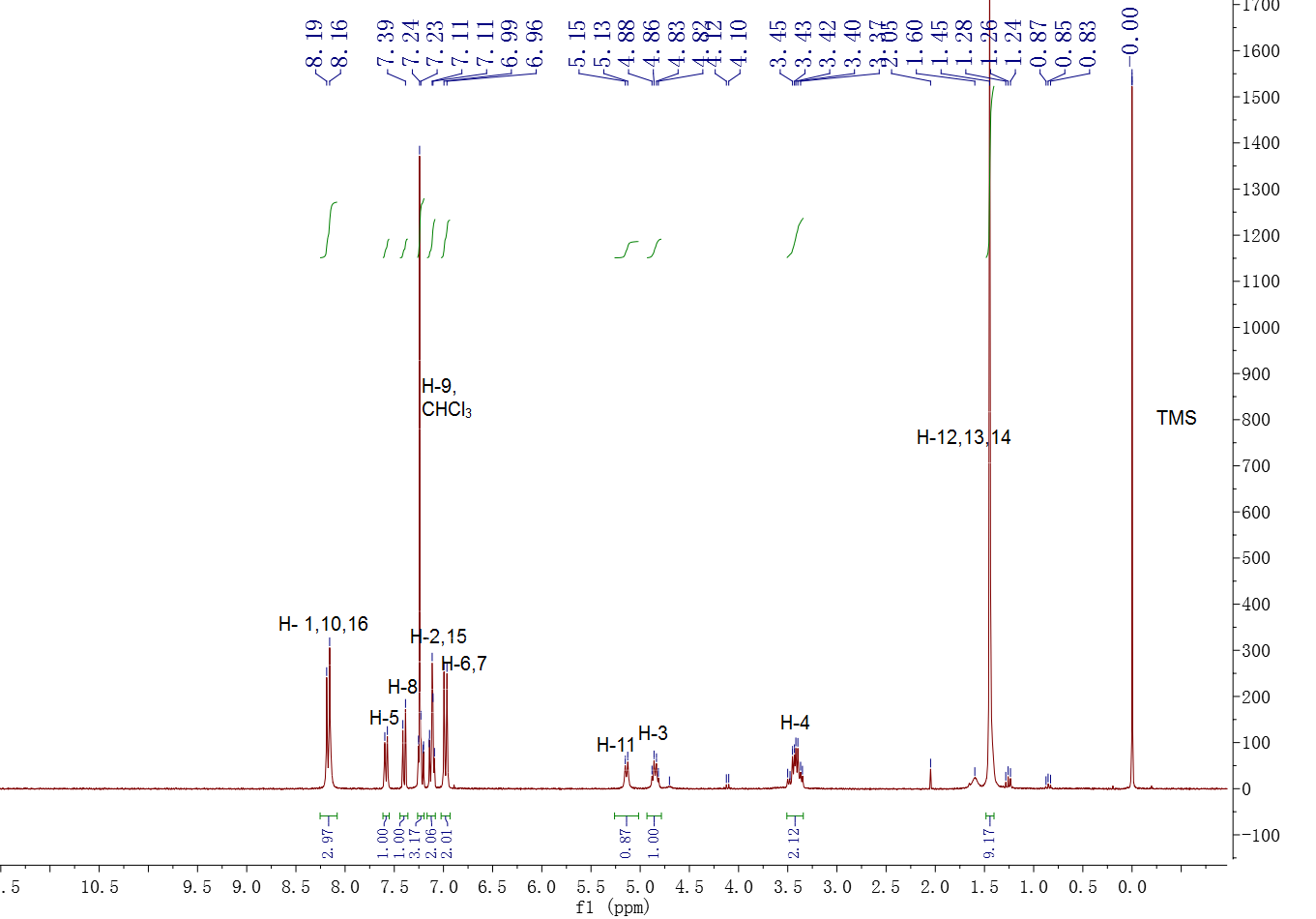


**Figure S7**. ^1^H NMR spectrum of ***L*-S_3_** in CDCl_3_.


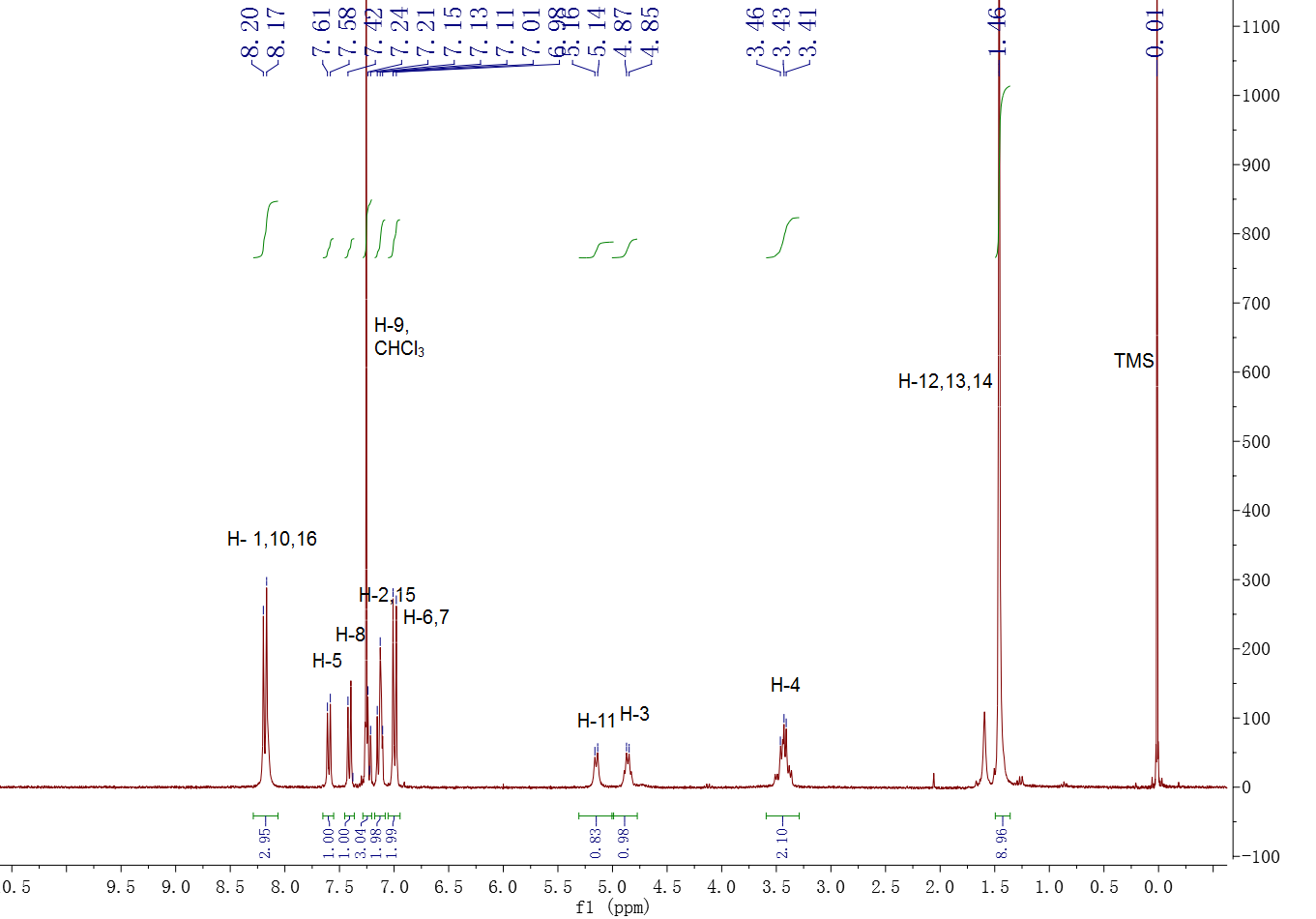


**Figure S8**. ^1^H NMR spectrum of ***D*-S_3_** in CDCl_3_.

**Figure S9**. Initial hydrolysis for **S_1_** (2.5 μM) promoted by different catalysts (50 μM) in HEPES buffer (pH = 7.2, 50 mM) containing 10% MeCN at (298 ± 0.1) K.

**Figure S10**. Initial hydrolysis for **S_2_** (2.5 μM) promoted by different catalysts (50 μM) in HEPES buffer (pH = 7.2, 50 mM) containing 10% MeCN at (298 ± 0.1) K.

**Figure S11**. Initial hydrolysis for **S_3_** (2.5 μM) promoted by different catalysts (50 μM) in HEPES buffer (pH = 7.2, 50 mM) containing 10% MeCN at (298 ± 0.1) K.


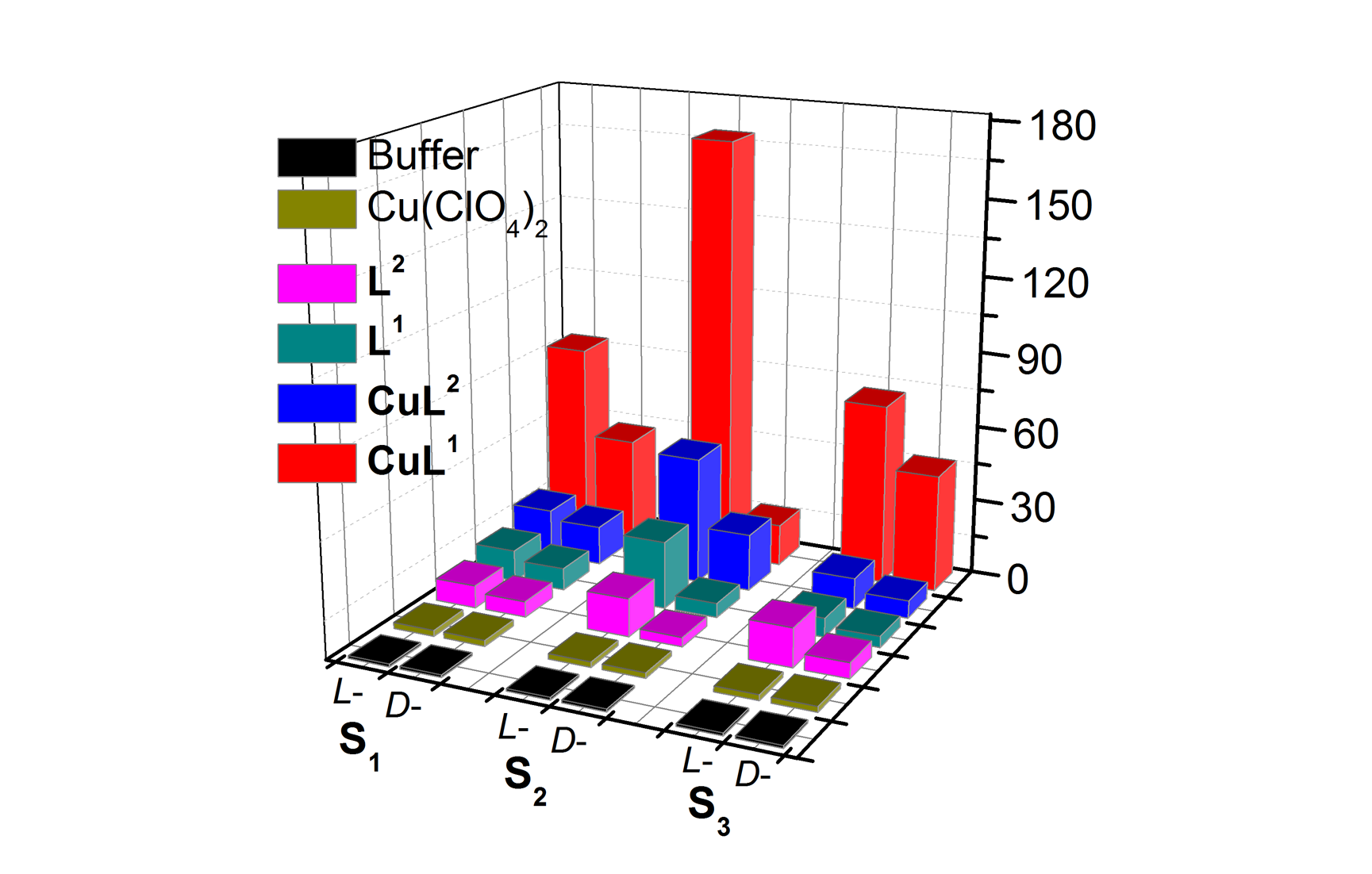


**Figure S12**. *k*_in_/*k*_uncat_ values for **S_1_**-**S_3_** (2.5 μM) promoted by different catalysts (50 μM) in HEPES buffer (pH = 7.2, 50 mM) containing 10% MeCN at (298 ± 0.1) K.

**Figure S13.** Hydrolysis of the single enantiomers of **S_2_** (2.5 μM) catalyzed by **CuL^1^** in a 10 % EtOH solution of HEPES buffer (pH 7.2, 50 mM) at (298 ± 0.1) K.


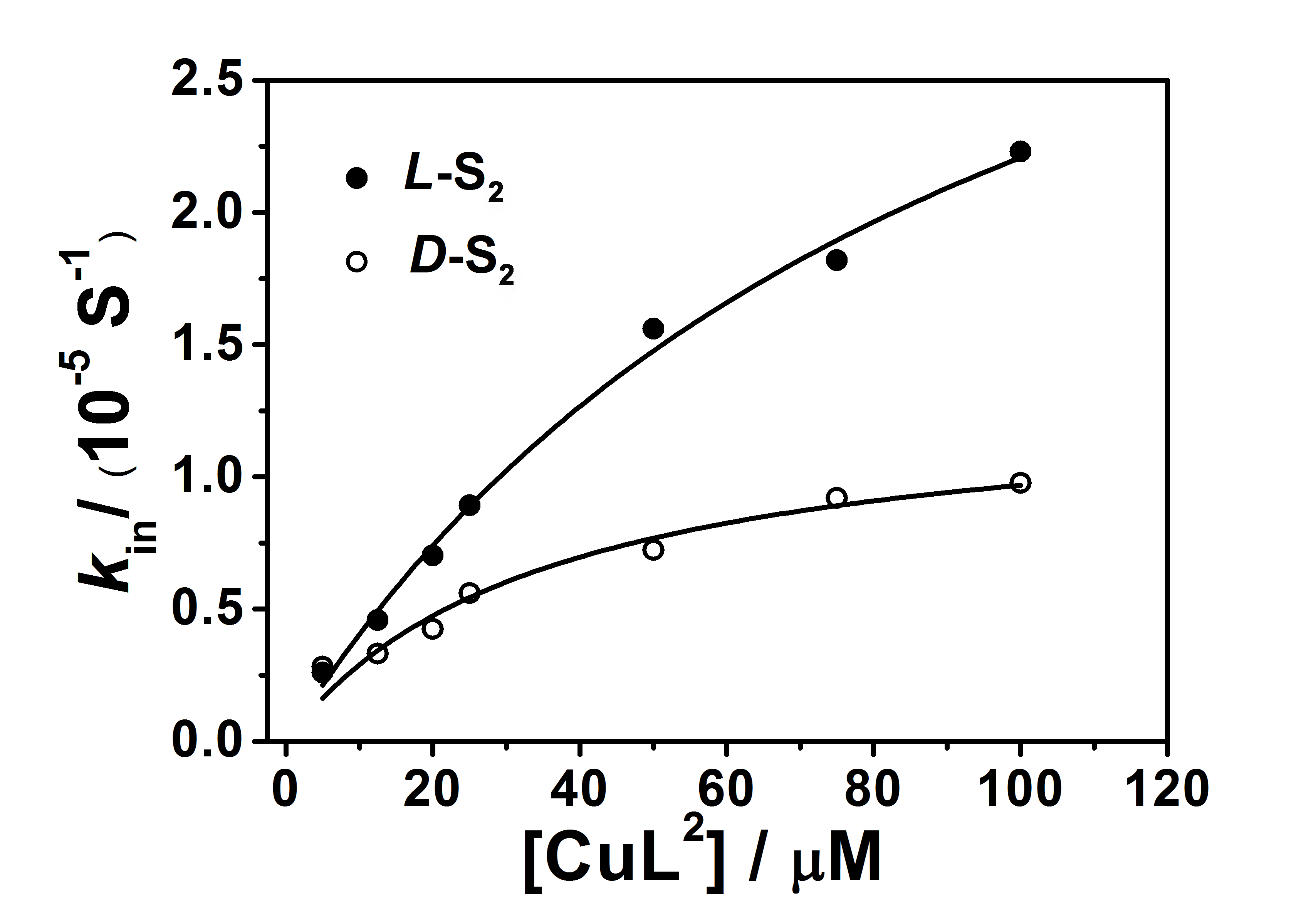


**Figure S14**. Michaelis–Menten kinetics for **S_2_** (2.5 μM) promoted by **CuL^2^** (5.0-100 μM) in HEPES buffer (pH 7.2, 50 mM) at (298 ± 0.1) K.


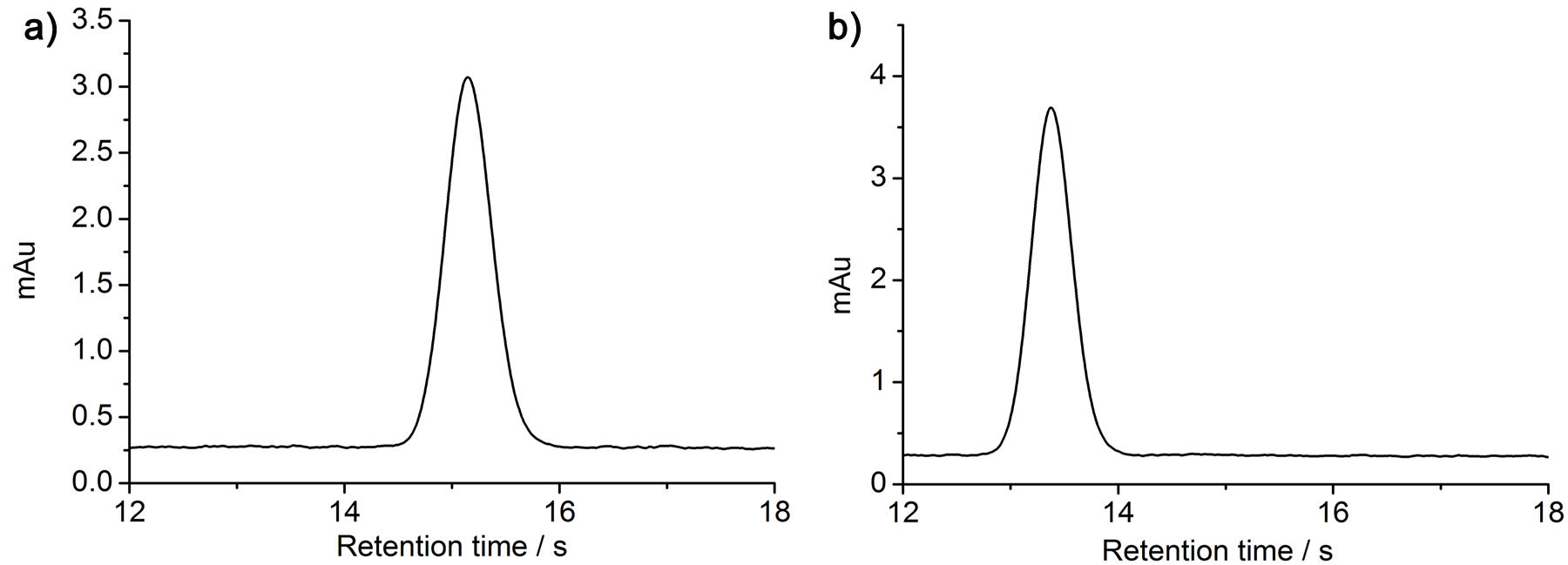


**Figure S15**. Chiral HPLC analysis results of a) ***L*-S_2_** and b) ***D*-S_2_**. Detection by 254 nm at room temperature, with elution solvent H_2_O/CH_3_CN/EtOH = 40/30/30, flow rate: 1.0 mL/min, column: CHIRALPAK® IC 250 × 4.6 mm.

**
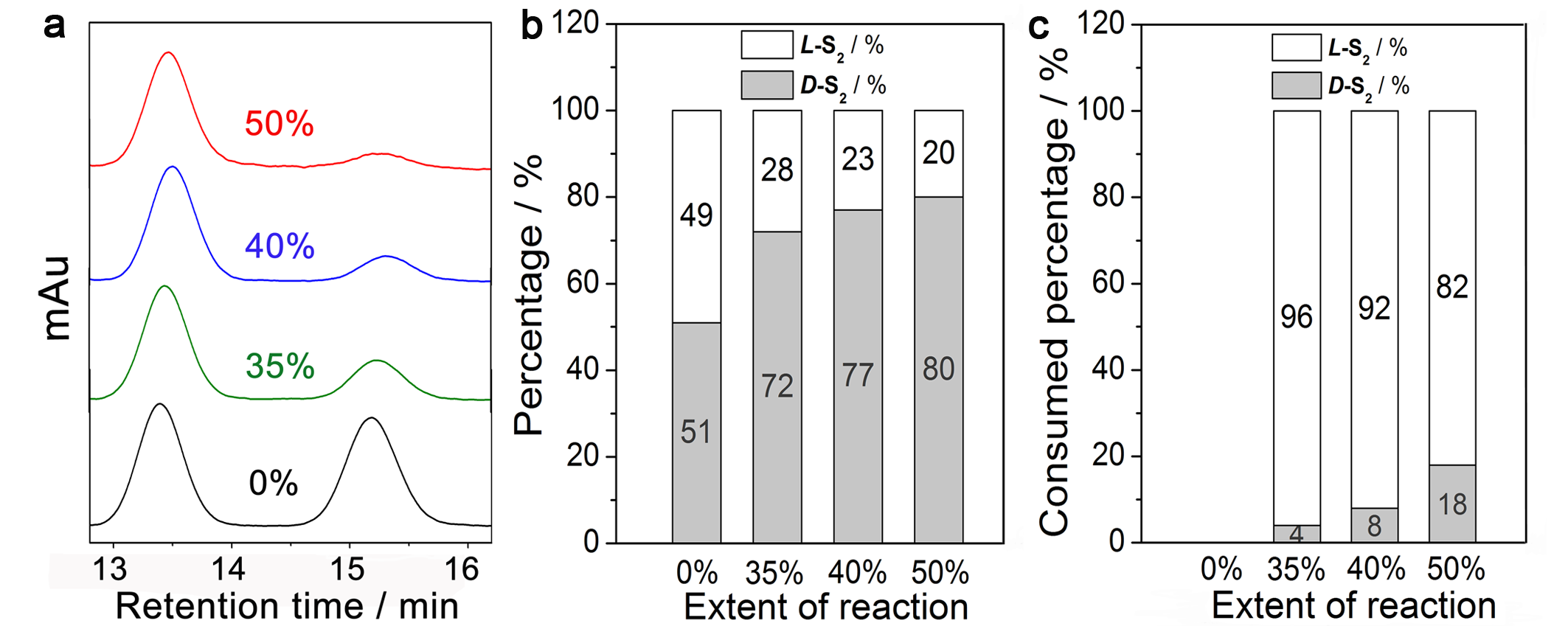
**

**Figure S16.** a) Chiral HPLC analyses for **S_2_** enantiomers in different conversions; b) ratios of ***L*-S_2_** and ***D*-S_2_** in different conversions; c) The consumed ratios of ***L*-S_2_** and ***D*-S_2_** worked out from b). Reaction condition: chiral HPLC analyses were performed at room temperature, monitored by a UV-Vis detector at 254 nm, with elution solvent H_2_O (0.1 % formic acid)/CH_3_CN/EtOH = 40/30/30, flow rate: 1.0 mL/min, column: CHIRALPAK® IC 250 × 4.6 mm.


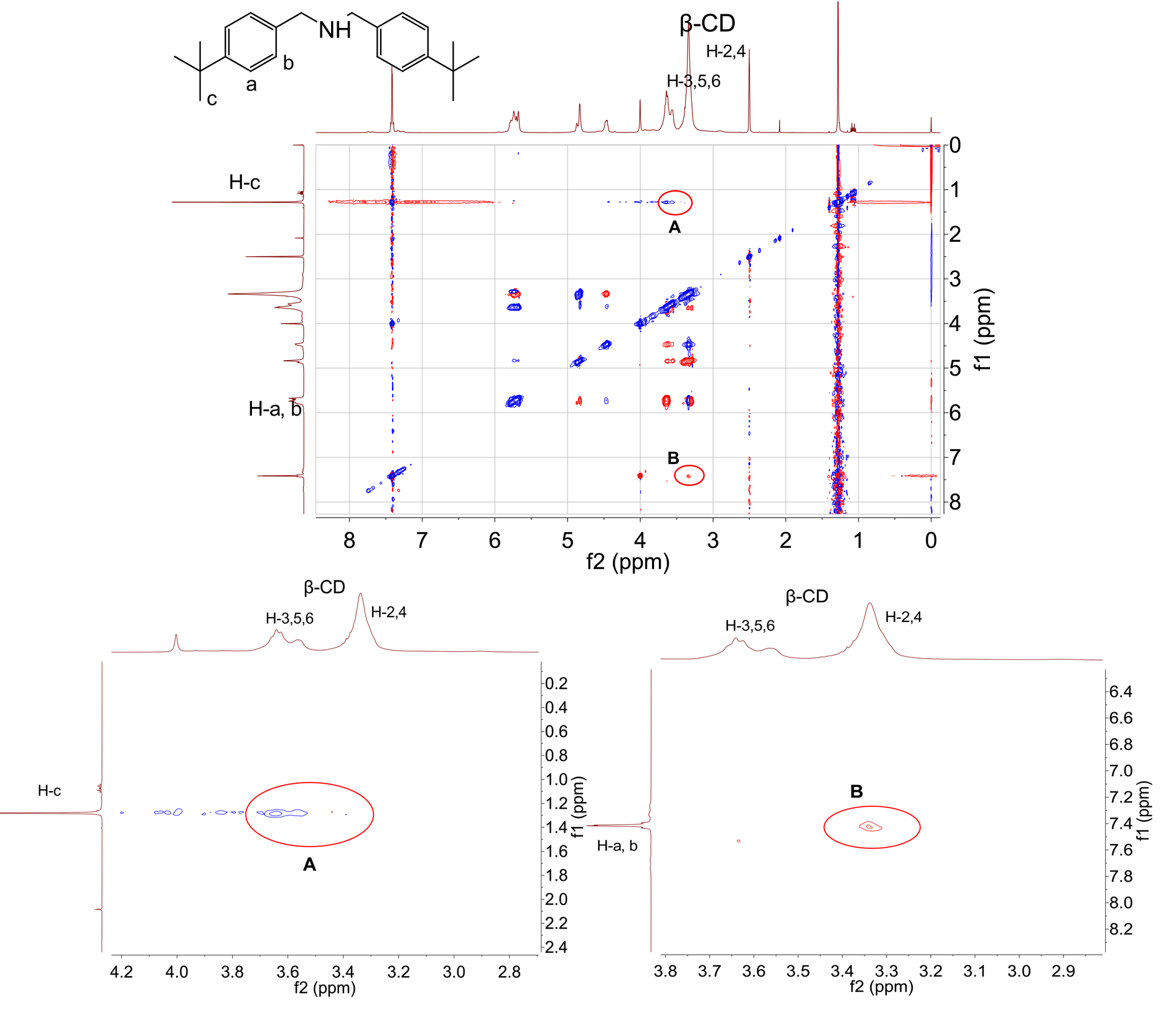


**Figure S17**. ROESY spectrum of **L^1^** (7.0 mM) with **DBBA** (20 mM) in *d*^6^-DMSO at 298 K.


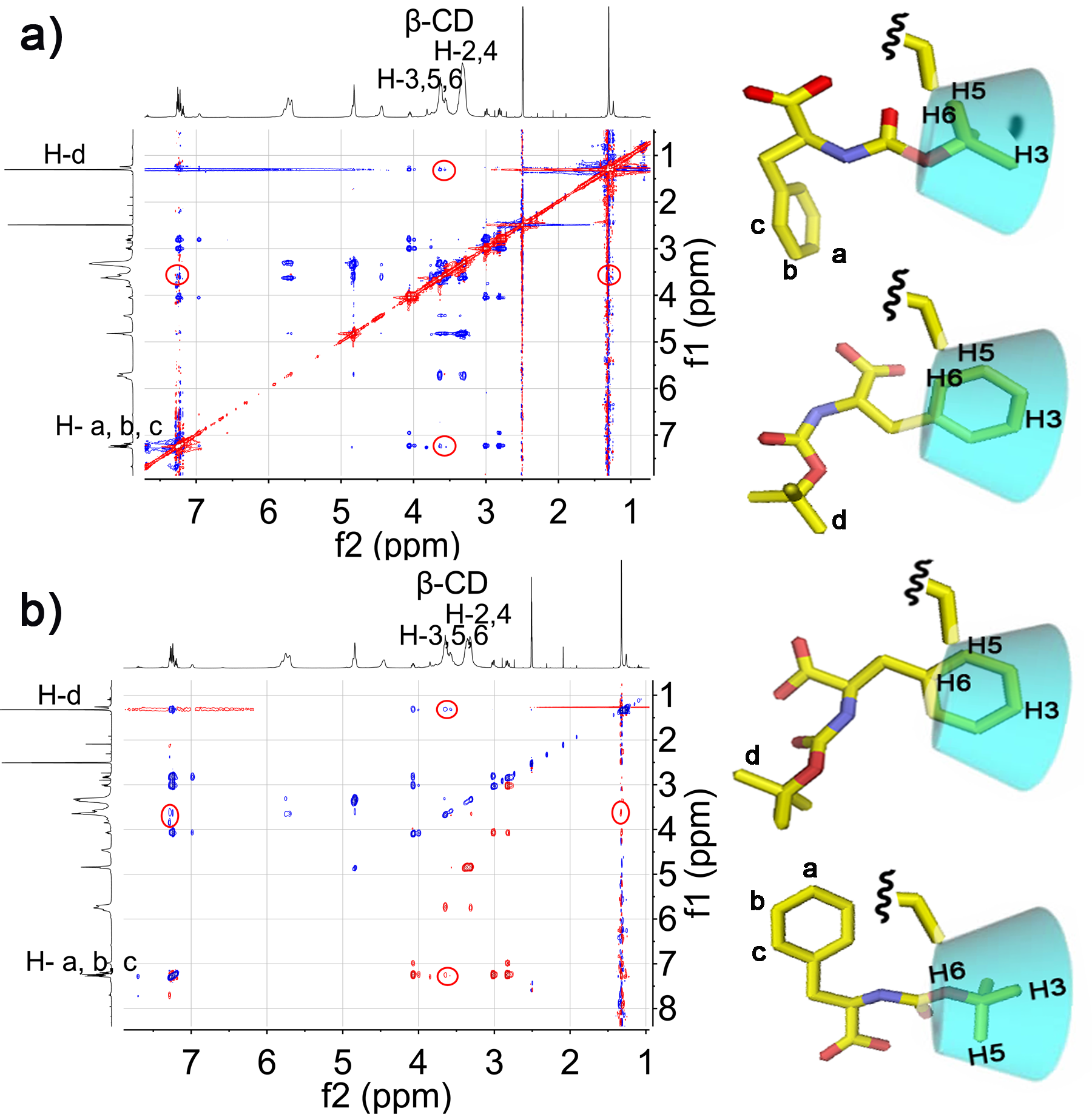


**Figure S18**. ROESY spectrum of **L^1^** (7.0 mM) with **a**) Boc-*L*-Phe-OH (20 mM) and **b**) Boc-*D*-Phe-OH (20 mM) in *d*^6^-DMSO at 298 K, and the possible binding manner with β-CD cavity.


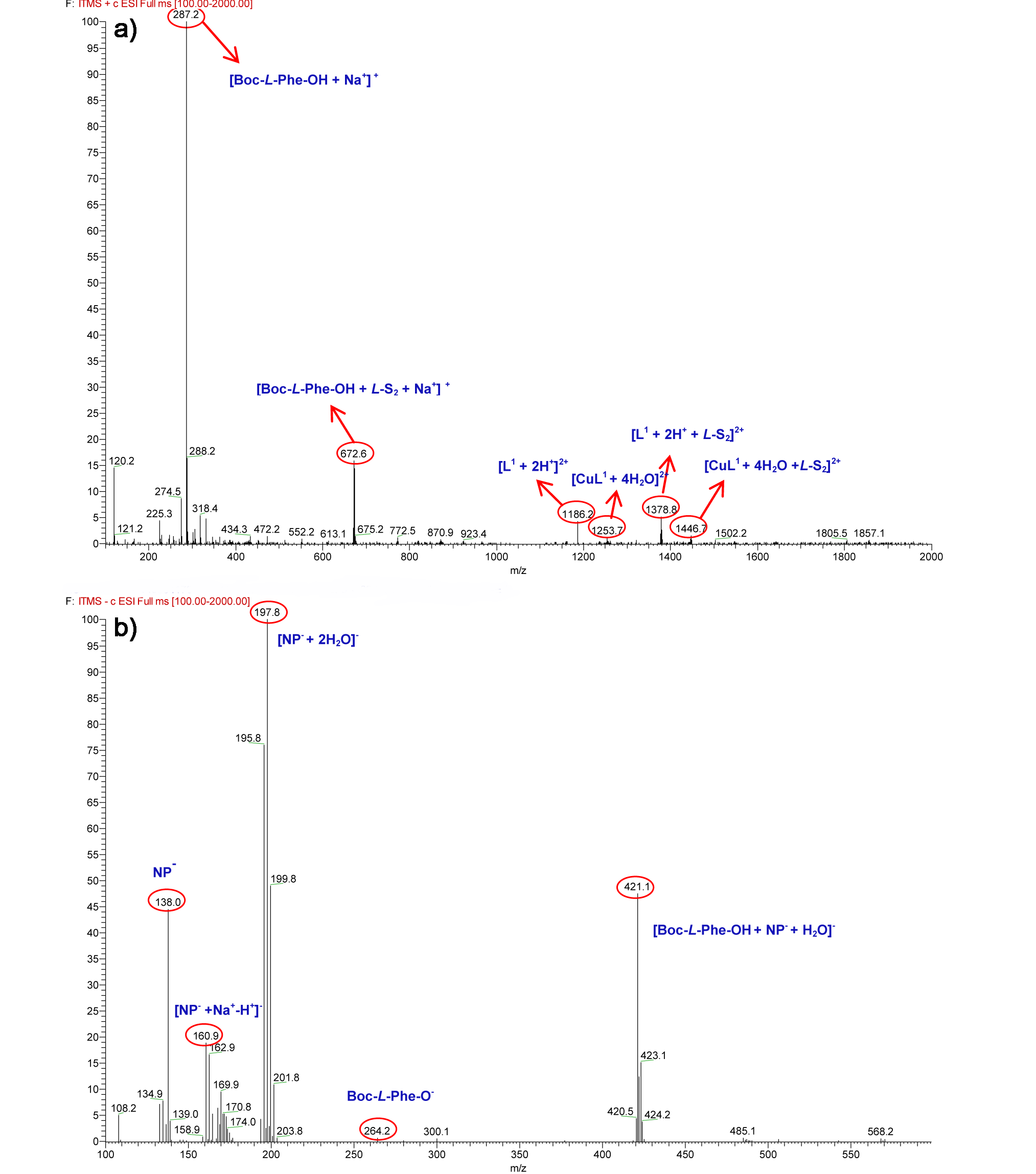


**Figure S19.** a) Positive-ion ESI-MS spectra of mixed solution of **CuL^1^** and ***L*-S_2_**. b) Negative-ion ESI-MS analysis of the ***L*-S_2_** hydrolysis products. Conditions: [**CuL^1^**] = 2.5 mM, [***L*-S_2_**] = 5.0 mM, H_2_O/CH_3_CN = 5/5 (v/v).


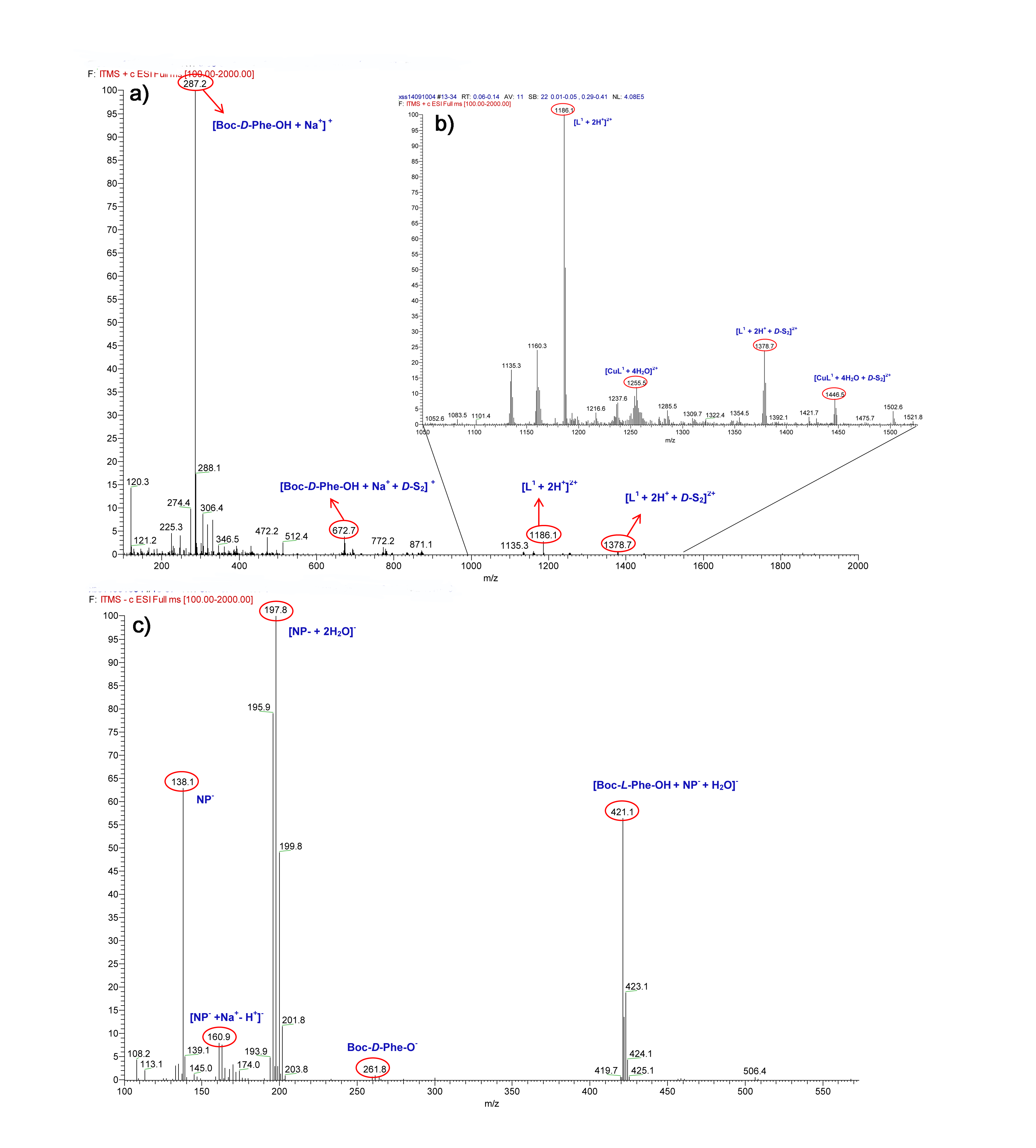


**Figure S20**. a) Positive-ion full range ESI-MS spectra of mixed solution of **CuL^2^** and ***D*-S_2_**. b) Expanded *m*/*z* = 1000-1500 of the full range spectra; c) Negative-ion ESI-MS analysis of the ***D*-S_2_** hydrolysis products. Conditions: [**CuL^2^**] = 2.5 mM, [***D*-S_2_**] = 5.0 mM, H_2_O/CH_3_CN = 5/5 (v/v).

**Table S1.** Kinetic parameters for ***L*-S_2_** and ***D*-S_2_** (2.5 μM) hydrolysis promoted by **CuL^2^** (5.0-100.0 μM) in a 10 % MeCN solution of HEPES buffer (50 mM, pH = 7.2) at (298 ± 0.1) K.

|  |  | *k*_cat_ (10^-5^ s^-1^) | *k*_cat_*^L^*/*k*_cat_*^D^* | *K*_m_ (×10^-2^ mM) |
| --- | --- | --- | --- | --- |
| **S_2_** | *L*- | 4.4 ± 0.4 | 3.3 | 9.8 ± 1.5 |
|  | *D*- | 1.3 ± 0.1 |  | 3.5 ± 0.9 |

**Table S2.** The kinetic parameters of **CuL^1^** to **S_2_** enantiomers with (*k*_in(i)_) or without (*k*_in_) **DBBA**. ^[a]^

|  | *k*_in_ (10^-5^ s^-1^) | *k*_in(i)_ (10^-5^ s^-1^) | *k*_in_/*k*_in(i)_ |
| --- | --- | --- | --- |
| ***L*-S_2_** | 2.9 | 2.1 × 10^-1^ | 13.7 |
| ***D*-S_2_** | 8.6 × 10^-1^ | 6.8 × 10^-1^ | 1.3 |

^[a]^ Reaction condition: HEPES buffer (pH 7.2, 50 mM) containing 10 % MeCN at (298 ± 0.1) K, [**CuL^1^**] = [**S_2_**] = [**DBBA**] = 10.0 μM.

**Table S3.** Initial rate constants for **S_2_** (2.5 μM) promoted by different complexes (50.0 μM) in HEPES buffer (pH 7.2, 50 mM) at (298 ± 0.1) K.

| Ligand | **S_2_** | *k*_in_ (10^-5^ s^-1^) | | | |
| --- | --- | --- | --- | --- | --- |
|  |  | **Cu^2+^** | **Zn^2+^** | **Co^2+^** | **Ni^2+^** |
| **L^1^** | *L*- | 5.4 | 1.4 | 7.5 × 10^-1^ | 1.7 |
|  | *D*- | 5.2 × 10^-1^ | 1.4 | 3.5 × 10^-1^ | 3.4 × 10^-1^ |
| **L^2^** | *L*- | 1.6 | 5.7 × 10^-1^ | 7.1 × 10^-1^ | 2.0 × 10^-1^ |
|  | *D*- | 7.2 × 10^-1^ | 6.1 × 10^-1^ | 2.5 × 10^-1^ | 3.1 × 10^-1^ |

**Table S4.** Initial rate constants for **S_2_** (2.5 μM) promoted by **CuL^1^** (50.0 μM) in HEPES buffer (pH 7.2, 50 mM) with 10 % cosolvent at (298 ± 0.1) K.

| Cosolvent | *k*_in_ (10^-5^ s^-1^) | | |
| --- | --- | --- | --- |
|  | ***L*-S_2_** | ***D*-S_2_** | *k*_in_*^L^*/*k*_in_*^D^* |
| CH_3_CN | 5.4 | 5.2 × 10^-1^ | 10.4 |
| EtOH | 6.2 | 5.9 × 10^-1^ | 10.5 |
